# Strain‐Modulated Engineering of High‐Entropy Vanadium‐Based Chalcogenide for Sustainable Water Oxidation

**DOI:** 10.1002/smll.73201

**Published:** 2026-03-22

**Authors:** Muhammad Zubair, Yongteng Qian, Kyung‐Ho Park, Dae Joon Kang

**Affiliations:** ^1^ Department of Physics Sungkyunkwan University Suwon Gyeonggi‐do Republic of Korea; ^2^ Pharmaceutical Engineering College Jinhua University of Vocational Technology Jinhua Zhejiang Province P. R. China; ^3^ Korea Advanced Nano Fab Center (KANC) Suwon Republic of Korea

**Keywords:** anode material, electrocatalysis, high entropy metal chalcogenide, oxygen evolution reaction, stability, strain engineering

## Abstract

High‐entropy metal chalcogenides (HEMC), stabilized by their high configurational entropy and multi‐element disorder, have emerged as promising materials for electrocatalysis. However, synthesizing high‐entropy sulfide catalysts via bottom‐up routes remains challenging due to the thermodynamic incompatibility of multiple metals, which promotes unwanted phase segregation and hinders controlled self‐assembly for optimal electrocatalytic performance. In this study, we tackle this challenge by systematically optimizing the solvothermal synthesis parameters, including solvent ratio, reductants, and stabilizers, to produce a single‐phase, strain‐engineered HEMC nanoflower/nanoflake (VMoFeCoNi)S_x_, as strain engineering has the potential to modify the adsorption process and enhance electrocatalytic activity. The Williamson–Hall analysis reveals a compressive micro strain of 0.67%, manifested as a blue shift of the (220) reflection (44.34° → 44.47°) and a slight lattice contraction relative to the control samples. The optimized HEMC‐based anode exhibits top‐level oxygen evolution reaction (OER) performance in alkaline media, achieving overpotentials of 210 mV and 250 mV at current densities of 50 mA cm^−2^ and 100 mA cm^−2^, respectively. Notably, it retains excellent OER stability with minimal degradation at 200 mA cm^−2^ over 120 h, demonstrating rapid reaction kinetics and durability at high current density, positioning it as a promising candidate for practical energy applications.

## Graphical abstract

High‐entropy metal chalcogenides (HEMC) incorporating five equimolar transition metals and sulfur were synthesized via an efficient solvothermal method. The resulting HEMC exhibits a single‐phase pyrite (Pa‐3) crystal structure, which was thoroughly characterized using advanced structural analysis techniques. As an anode material, the HEMC demonstrates exceptional electrocatalytic properties, driven by enhanced electronic conductivity and an increased density of catalytically active sites.

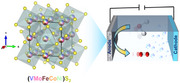

## 1 Introduction

Metal chalcogenides have garnered significant attention for their versatile applications in electronics [1], photovoltaics [2], thermoelectrics [3], and electrocatalysis [4], positioning them as critical materials for advancing renewable energy technologies to mitigate reliance on fossil fuels and address environmental challenges [5]. While binary metal chalcogenides (M_x_S_y_) have been extensively studied for electrochemical purposes, their practical utility is often hindered by limited stability under oxidative conditions, manifesting as thermodynamic instability, structural degradation, and catalyst detachment from the conductive substrate used as an electrode [6, 7]. Additionally, unary, binary, and ternary M_x_S_y_ systems offer limited compositional flexibility, restricting their tunability for optimized catalytic performance [8]. Recent advancements in multi‐metallic chalcogenides, such as ternary and quaternary systems (e.g., Cu_2_CdSnS_4_ for photocatalytic applications), have demonstrated enhanced performance through synergistic multi‐metallic interactions [9]. The catalytic performance of metallic surfaces is strongly governed by lattice distortions arising from atomic mismatches in alloyed systems. Such distortions alter surface interatomic distances relative to the bulk, inducing localized compressive or tensile strain that modifies the electronic structure and adsorption energetics of reaction intermediates. In particular, compressive strain shifts electronic energy levels near the Fermi surface, enabling optimized intermediate binding and enhanced electrocatalytic activity, thereby establishing strain engineering as an effective strategy for catalyst design [10, 11].

Inspired by the success of high‐entropy alloys [12], oxides [13], and halides [14], which exhibit improved stability and catalytic efficiency, high‐entropy metal chalcogenides (HEMC) have emerged as a promising class of materials for thermoelectric and electrocatalytic applications [15]. HEMCs are characterized by the incorporation of five or more equimolar metallic elements within a sulfide framework, stabilized by high configurational entropy that promotes a single‐phase crystalline structure. This entropy‐driven stabilization, distinct from conventional enthalpy‐dominated materials, results in a single diffraction pattern indicative of a uniform polymorph [16]. Although a universal definition of high‐entropy materials remains elusive, a configurational entropy (S_conf_) greater than 1.5R (where R is the general gas constant) is often proposed as a threshold for high‐entropy alloys [17]. The compositional versatility of HEMC enables precise tuning of catalyst‐adsorbate interactions, aligning with the Sabatier principle to optimize the adsorption of reaction intermediates and enhance catalytic activity [18]. The high‐entropy structure further enhances phase stability, mitigating degradation during oxygen evolution reaction (OER) processes [19]. However, synthesizing high‐quality HEMC with uniform phase and optimal electrocatalytic properties remains a significant challenge.

High‐entropy alloys (HEAs) have emerged as promising electrocatalytic materials due to their tunable surface chemistry and compositional diversity, enabling enhanced functionality for energy conversion applications. Therefore, optimizing the hydrogen binding energy (HBE) requires precise modulation of the catalyst's electronic structure and d‐band center, achieved through strategic incorporation of high‐valent metals such as vanadium, chromium, molybdenum, or tungsten [20]. These dopants effectively regulate the adsorption behavior of water and reaction intermediates, significantly enhancing the electrochemical performance of electrode materials. For instance, Mei et al. developed a molybdenum‐coordinated HEA (FeCoNiMo) and demonstrated, through methanol molecular probe experiments and X‐ray photoelectron spectroscopy, that electron transfer from molybdenum to iron, cobalt, and nickel weakens OH^*^ binding, thereby improving oxygen evolution reaction (OER) efficiency [21]. Similarly, Sivanantham et al. explored the role of vanadium in a CuCoNiFeMn HEA, utilizing density functional theory (DFT) calculations to show that vanadium incorporation reduces the Gibbs free energies for water dissociation and hydrogen adsorption, leading to accelerated hydrogen evolution reaction (HER) kinetics [22]. These findings highlight the potential of incorporating high‐valent metals in HEAs to enhance electrocatalytic activity for sustainable energy applications. Among the diverse array of transition metals, vanadium stands out as an ideal dopant due to its versatile multivalent states (+2 to +5), which enable dynamic modulation of the electronic structure through synergistic interactions with the host material. Its earth‐abundant nature and cost‐effectiveness further enhance its appeal for catalytic applications [23]. Vanadium incorporation significantly enhances intrinsic catalytic performance by increasing the density of active sites and improving structural adaptability, thereby optimising the electrocatalytic properties of the host [24]. While vanadium's integration into oxides and hydroxides has been well‐documented for improving catalytic efficiency through pronounced electronic and structural synergies [25], its application within high‐entropy chalcogenide frameworks remains largely unexplored.

The synthesis of entropy‐stabilized high‐entropy metal chalcogenides remains a nascent field, with only a handful of studies reporting successful preparation. Existing methods often rely on complex, energy‐intensive processes that involve prolonged procedures, specialized substrates, and extreme thermal conditions. For instance, one study employed carbonized wood as a support for (CrMnFeCoNi)Sx, utilizing pulsed thermal decomposition of metal salts and thiourea, which required a 6‐hour drying phase, high‐temperature annealing (∼1650 K) for brief durations (∼55 ms), and rapid quenching [26]. More recently, Cai et al. developed a high‐entropy sulfide encapsulated within porous carbon nanofibers through a multi‐step electrospinning and annealing approach [27]. Another investigation reported the preparation of high‐entropy NiCoMnCrVSe2 nanoflakes anchored on graphene supports via a microwave‐assisted solvothermal technique [28]. These efforts highlight the challenges and diversity in synthetic strategies for high‐entropy chalcogenides, underscoring the need for more efficient and scalable methods to unlock their potential in electrocatalytic applications.

This study explores the untapped potential of strain‐modulated engineering of high‐entropy chalcogenide systems for oxygen evolution reaction (OER) catalysis by utilizing the mismatch of atomic and bonding configurations within their entropy‐stabilised coordination. Modulating lattice strain offers a powerful means to tailor the electronic structure of electrocatalysts, thereby optimizing the binding strength of intermediates and enhancing catalytic performance. We hypothesized that incorporating vanadium into a multi‐metallic sulfide matrix comprising molybdenum, iron, cobalt, and nickel would generate a strain, thereby tailoring the electronic environment with a high density of catalytic sites, driven by synergistic interactions among the constituent elements. Here, we introduce an efficient two‐step solvothermal conversion/reduction strategy to synthesize a single‐phase, vanadium‐integrated HEMC, denoted as (VMoFeCoNi)Sx. The catalyst design involved careful selection of solvents, stabilizing agents, and 3d transition metals (Fe, Co, Ni) and molybdenum, a non‐3d high‐valent metal, for their complementary catalytic properties. Iron, cobalt, and nickel were chosen for their ability to enhance electrocatalytic activity through ligand effects, while molybdenum was selected for its noble‐metal‐like catalytic characteristics. This strategic compositional engineering leverages high‐entropy effects to restrict elemental diffusion, while simultaneously optimizing the electronic structure to modulate cohesive and adsorption energies, thereby enhancing resistance to elemental dissolution. The resulting (VMoFeCoNi)Sx anode with 0.67% compression micro strain exhibits a large electrochemically active surface area and a superaerophobic surface, delivering OER performance in alkaline media, with a low overpotential of 210 mV at 50 mA cm^−2^ and stable operation over 120 h at 200 mA cm^−2^. This performance surpasses many leading transition metal sulfides and high‐entropy alloy electrocatalysts, positioning (VMoFeCoNi)Sx as a highly promising material for efficient and durable water oxidation in sustainable energy applications.

## 2 Results and Discussion

### 2.1 Preparation

**SCHEME 1:**
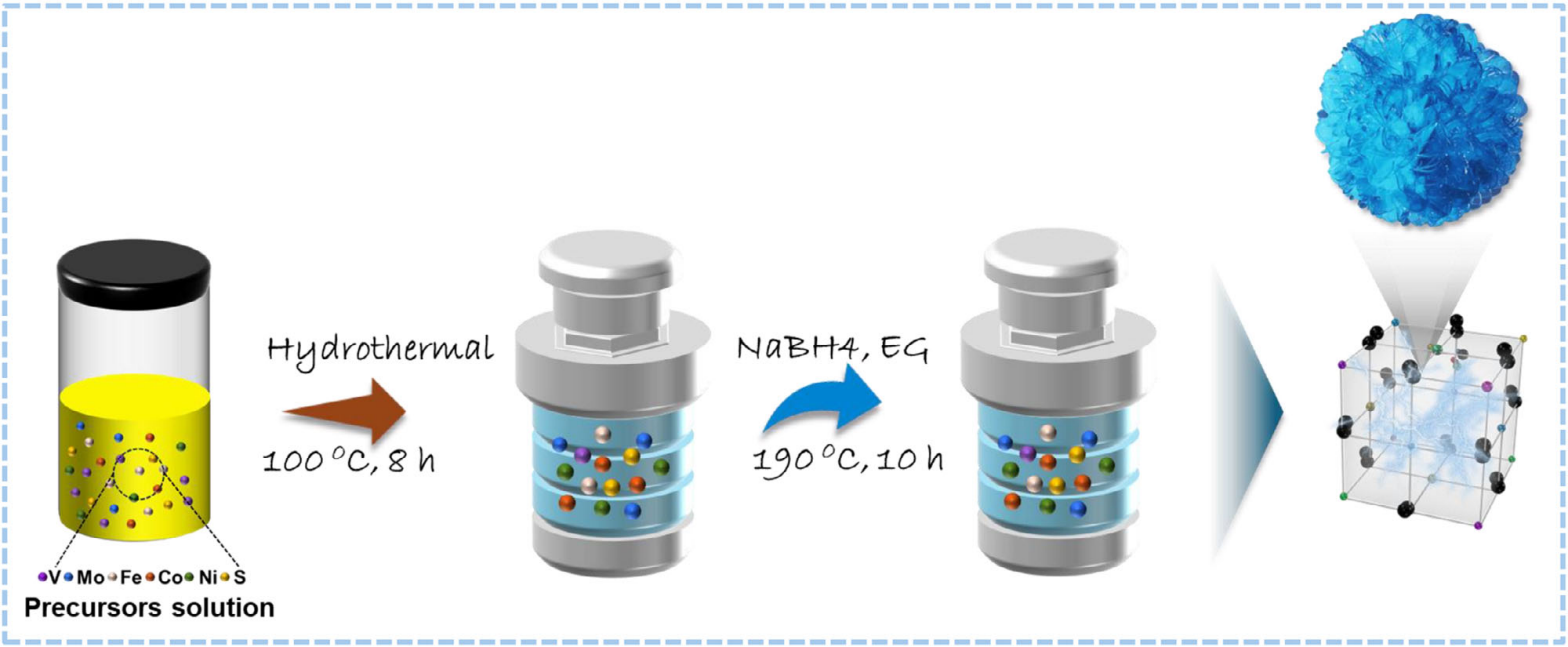
A schematic representation illustrates the synthesis of high‐entropy metal chalcogenide (HEMC) through an optimized two‐step solvothermal process.

The role of configurational entropy in dictating the structural and functional properties of disordered materials is a critical area of investigation. To achieve pronounced synergistic effects among constituent elements, the distribution of active species must be highly randomized, thereby maximizing configurational entropy. Based on the configurational entropy equation $\Delta Sconf=-R\sum_{i=1}^{n}x\mathrm{iln}xi$ where xi represents the molar fraction of each component, the entropy is maximized when all components are present in equimolar proportions (x i = 1/n). However, the precise synthesis of nanoscale high‐entropy metal chalcogenide (HEMC) with controlled morphologies and dimensions poses significant challenges due to the diverse physicochemical properties of the constituent metals. Notably, the fabrication of HEMC nanosheets and nanoflower arrays remains underexplored. In this study, we report a facile two‐step solvothermal synthesis of (VMoFeCoNi)Sx strain‐modulated HEMC nanoflower arrays through an optimized conversion/reduction approach, using ethylene glycol as the reducing solvent and PVP as the growth‐promoting agent (molecular template), as detailed in the Experimental Section (Scheme 1). To achieve uniform and morphologically diverse high‐HEMC, PVP was introduced as a stabilizing agent. PVP adsorbs onto the surfaces of growing crystals, thereby lowering the interfacial energy between the crystal and the solution, and promoting nucleation. Additionally, the selective surface adsorption of PVP suppresses the formation of irregular or defective crystalline structures, enabling the controlled growth of well‐defined, homogeneous HEMC architectures [29]. This “capping” effect prevents the strained crystallites from growing too large or relaxing their surface strain. Ethylene glycol (EG), a diol‐based reducing solvent, forms weak coordination complexes with metal ions due to its two hydroxyl groups, as explained by molecular orbital theory, which indicates stability in its monoanionic form. The limited reactivity of EG with metal ions stems from its low‐energy highest occupied molecular orbital (HOMO). However, when heated near its boiling point, the hydrogen bonds within EG are disrupted, elevating it to a high‐energy state with enhanced reactivity [30]. The EG viscosity and reducing power control the nucleation rate. Furthermore, the strain modulation was achieved by optimizing the reaction kinetics to favor entropy‐driven mixing over enthalpy‐driven segregation. The ratio of EG to water is critical in this work. An optimized EG ratio ensures simultaneous nucleation rather than subsequent precipitation. This rapid, simultaneous locking of atoms with different radii prevents the lattice from relaxing into a lower‐energy, segregated state, and effectively “trapping” the high‐entropy induced strain. Moreover, the reaction temperature and duration regulate the balance between crystallization and strain relaxation because excessive thermal energy would allow atomic diffusion, leading to phase segregation and strain relief. Our optimized solvothermal parameters preserve the metastable high‐strain state by terminating crystal growth before thermodynamic equilibrium (relaxation) is reached.

### 2.2 Characterization

**FIGURE 1:**
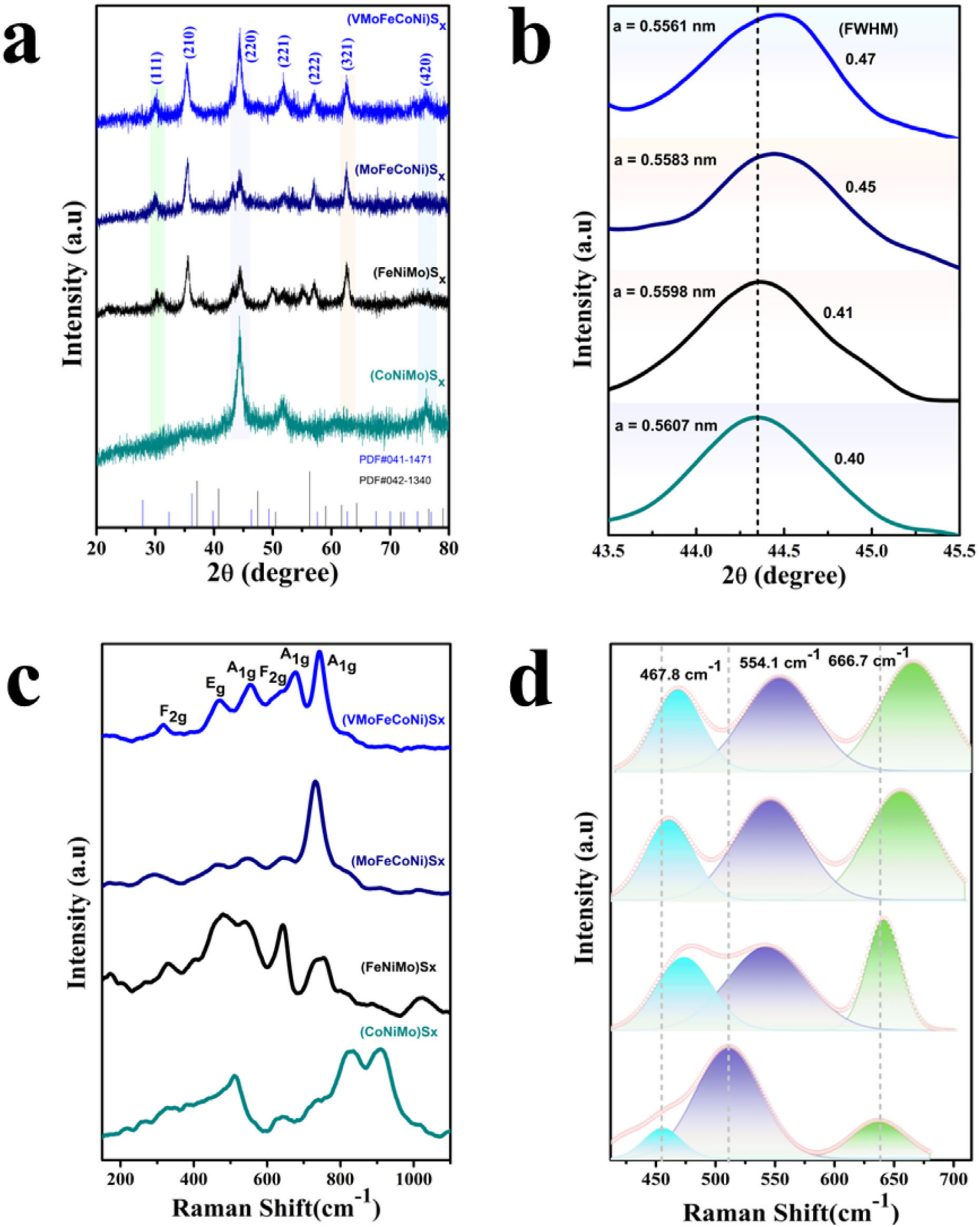
Structural and optical evaluation of HEMC and control samples. (a) pXRD patterns of the as‐synthesized (VMoFeCoNi)S_x_ along with control samples, and (b) Enlarged view of 220 diffraction plan, (c) Raman spectra of (VMoFeCoNi)S_x_ along with control samples, showing the presence of symmetric bending and stretching vibration, and (d) Raman peak fitting depicting the blue shifting.

A primary criterion for evaluating high‐entropy materials is the presence of a single‐phase crystalline structure, verified through X‐ray diffraction (PXRD) analysis. The (VMoFeCoNi)S_x_ exhibits a characteristic cubic pyrite structure (PDF#00‐041‐1471, 00‐042‐1340) with the Pa‐3 space group [31], evidenced by seven distinct diffraction peaks at 30.0°, 35.3°, 44.4°, 51.7°, 56.9°, 62.5°, and 76.1°, corresponding to the (111), (210), (220), (221), (222), (321), and (420) planes, respectively (Figure 1a). These results confirm the successful synthesis of a single‐phase (VMoFeCoNi)S_x_ HEMC. Notably, the (220) peak of (VMoFeCoNi)S_x_ displays a pronounced blue shift (44.34°→ 44.47°) relative to control samples (Figure 1b), while the (210) peak also exhibits a blue shift, indicative of significant compressive lattice strain induced by the high‐entropy alloy formation [32]. This lattice strain results in structural distortion, manifesting as peak broadening in the XRD patterns, characterized by an increased full width at half maximum (FWHM) as shown in Figure 1b, likely attributable to localized charge density accumulation. The lattice distortion is further quantified through Williamson‐Hall analysis [33], as depicted in Figure S1, where the micro strain of HEMC (0.67%) is significantly higher than that of the control samples, thereby further verifying the aforementioned conclusions. The Williamson‐Hall analysis confirms this compressive strain, consistent with the Raman blue shifts that reflect stronger M─S bonding. Additionally, an observed decrease in the lattice constant supports the distortion phenomenon in the final material [34]. Table S1 summarizes the crystallite size, dislocation density, and micro‐strain values for the HEMC alongside its ternary and quaternary reference samples. The HEMC exhibits significantly higher micro strain compared to the reference materials, a phenomenon driven by the incorporation of high‐valent metal atoms. These atoms introduce lattice distortions, inducing atomic‐level strain that modifies the local coordination environment and increases microstrain within the crystalline structure. Additionally, the HEMC exhibits a higher dislocation density compared to the control samples, which is attributed to enhanced lattice perturbations. These distortions arise from the accommodation of high‐valent atoms, which possess differing atomic radii, thereby increasing the density of structural defects within the crystal lattice [35].

To authenticate the single‐phase nature and lattice distortion induced by the high‐entropy effect, Raman spectroscopy was employed due to its high sensitivity to structural defects and compositional heterogeneity (Figure 1c). In a cubic lattice, first‐order Raman active modes are typically absent [36]. However, the disordered cubic structure of the HEMC, resulting from the random distribution of atomic species, induces Raman‐active vibrational modes, including stretching (A_1g_ and E_g_) and bending (F_2g_) modes [37]. The (VMoFeCoNi)S_x_ HEMC exhibits six prominent Raman peaks: one E_g_ mode at 467.8 cm^−1^ corresponding to asymmetric twisting of transition metal (TM) cations, three A_1g_ modes at 554.1 cm^−1^, 666.7 cm^−1^, and 744.6 cm^−1^ associated with stretching vibrations, and two F_2g_ modes at 316.9 cm^−1^ and 637.6 cm_‐1_ attributed to M–S bending vibrations. The presence of E_g_ and F_2g_ modes provides direct evidence of lattice distortion within the lattice framework, a hallmark of high‐entropy materials [38]. The incorporation of high‐valent metal cations and sulfur anions induces a pronounced shift of Raman bands to higher wavenumbers [39], reflecting adjustments in vibrational force constants (Figure 1d). Detailed peak fitting was performed on the spectra of HEMC and control samples, revealing distinct vibrational modes. The fitted peak positions (E_g_ and A_1g_), full‐widths half maximum (FWHM), and fitting quality (R^2^) are summarized in Table S2, showing pronounced blue‐shift as compared to pyrite FeS_2_ and CoS_2,_ along with the observed broadening (FWHM). This spectrally observed lattice strain, which systematically increases with compositional complexity, aligns well with the XRD peak shifts and broadening. Additionally, variations in the integral intensities of these bands were observed, with symmetric vibrational modes exhibiting higher intensities than asymmetric ones, consistent with Raman spectroscopy's greater sensitivity to symmetric vibrations. This spectral behavior is attributed to cation disorder and localized structural distortions at the atomic level, driven by the high‐entropy configuration. These distortions enhance the asymmetry of M–S stretching vibrations, further amplifying peak shifts and intensity changes in the Raman spectra, confirming the structural complexity and uniformity of the HEMC.

**FIGURE 2:**
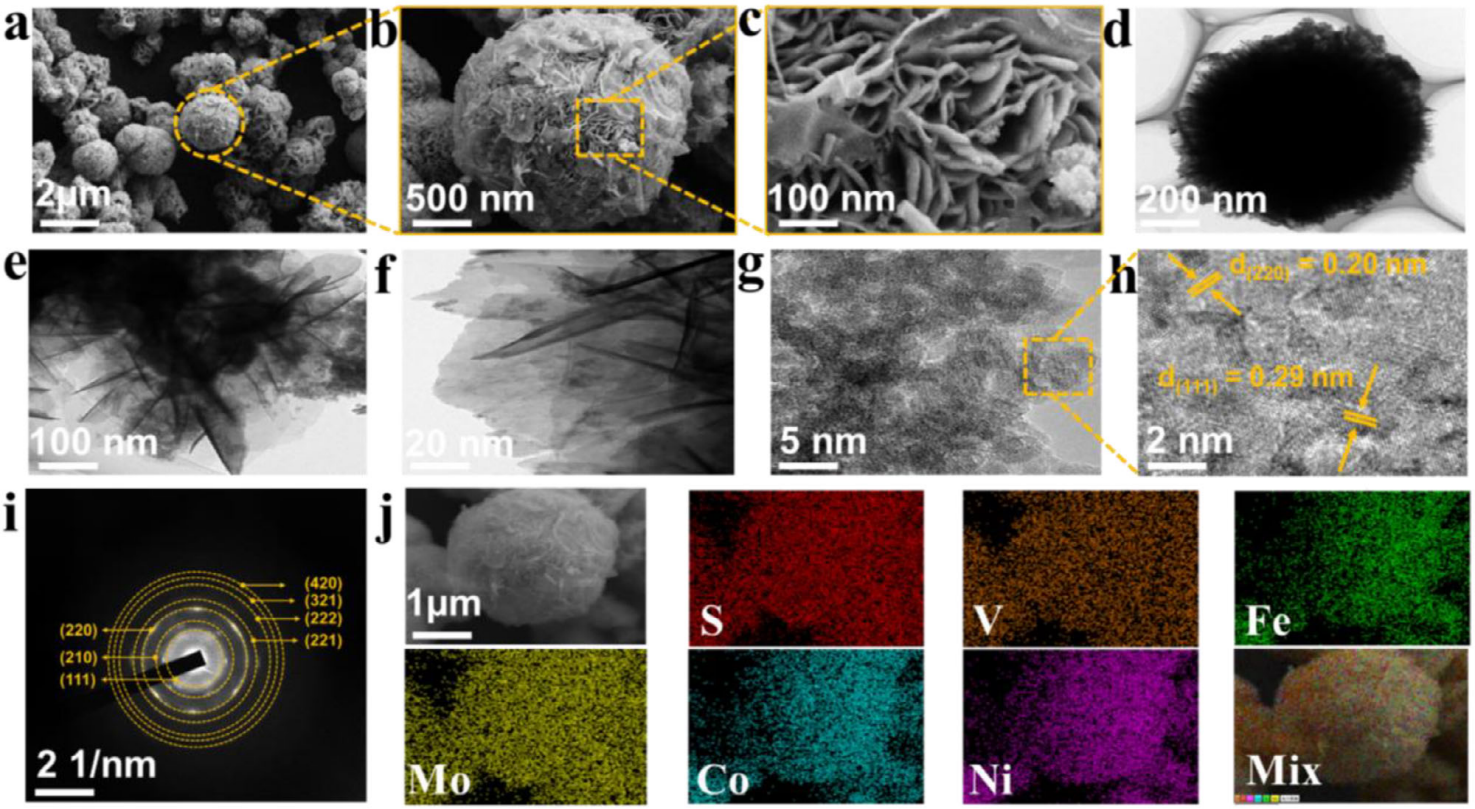
Morphological characterization of nanoflower/nanosheets HEMC. (a–c) Representative SEM images at different magnifications representing nanoflower/nanosheets morphology; the marked yellow circle and rectangle in the panels (a and b) demonstrate the projection of the selected area, highlighting the porous nanosheets nature, (d–f) TEM images showing the well‐defined nanosheets structure, (g, h) HRTEM images of nanoflower/nanosheet displaying well‐resolved lattice fringes, (i) Selected area electron diffraction (SAED) showing well‐indexed diffraction rings, (j) elemental mapping of HEMC, representing the uniform distribution of V, Mo, Fe, Co, Ni, and S within single phase matrix without any significant impurities.

The surface morphology and topography of the synthesized HEMC were analysed using scanning electron microscopy (FE‐SEM) and transmission electron microscopy (TEM). High‐resolution SEM images at varying magnifications (Figure 2a–c) demonstrate a textured surface comprising interconnected nanoflake and nanoflower arrays, forming a cohesive structural network that maximizes catalytic site accessibility, thereby enhancing electrocatalytic efficiency. The high surface area is particularly advantageous for water electrolysis, as it ensures sustained catalytic activity by maintaining a high density of accessible sites, even in the presence of intermediate species that may irreversibly adsorb to some sites. The HEMC nanosheets, with an average thickness of less than 10 nm, form a highly interconnected framework that facilitates efficient electron and ion transport by minimizing diffusion barriers. Moreover, SEM imaging reveals macro voids within the structure (Figure 2c), contributing to a hydrophilic‐aerophobic surface that enhances water electrolysis performance. High‐resolution transmission electron microscopy (TEM) analysis (Figure 2d–f) further validates the well‐defined hierarchical nanoflower architecture, comprising interconnected 2D nanosheet arrays that assemble into a 3D open‐framework network, thereby maximizing accessible surface area. Notably, as illustrated in Figure 2g,h and Figure S2, the high‐resolution TEM (HRTEM) micrographs reveal lattice fringes at 0.29 nm and 0.20 nm, corresponding to the (111) and (220) planes, respectively, which match the XRD peaks at 30° and 44.4°. Additionally, the selected‐area electron diffraction (SAED) pattern (Figure 2i) exhibits sharp spots and rings, indicative of high crystallinity. These findings align with the XRD results (Figure 1a), confirming a single‐phase high‐entropy alloy (HEA) free of impurities or segregation. Energy‐dispersive X‐ray spectroscopy (EDS) coupled with SEM/TEM reveals the homogeneous distribution of vanadium, molybdenum, iron, cobalt, nickel, and sulfur, sharing the identical spatial distribution on a micrometer scale (Figure 2j; Figure S3), with no detectable impurities, confirming the chemical uniformity characteristic of HEMC. The EDS spectra further validate the stoichiometric precision of the constituent elements (Figure S4). Comparative morphological analysis, EDS mapping, and spectra of reference samples were systematically investigated, as presented in Figures S5–S10.

**FIGURE 3:**
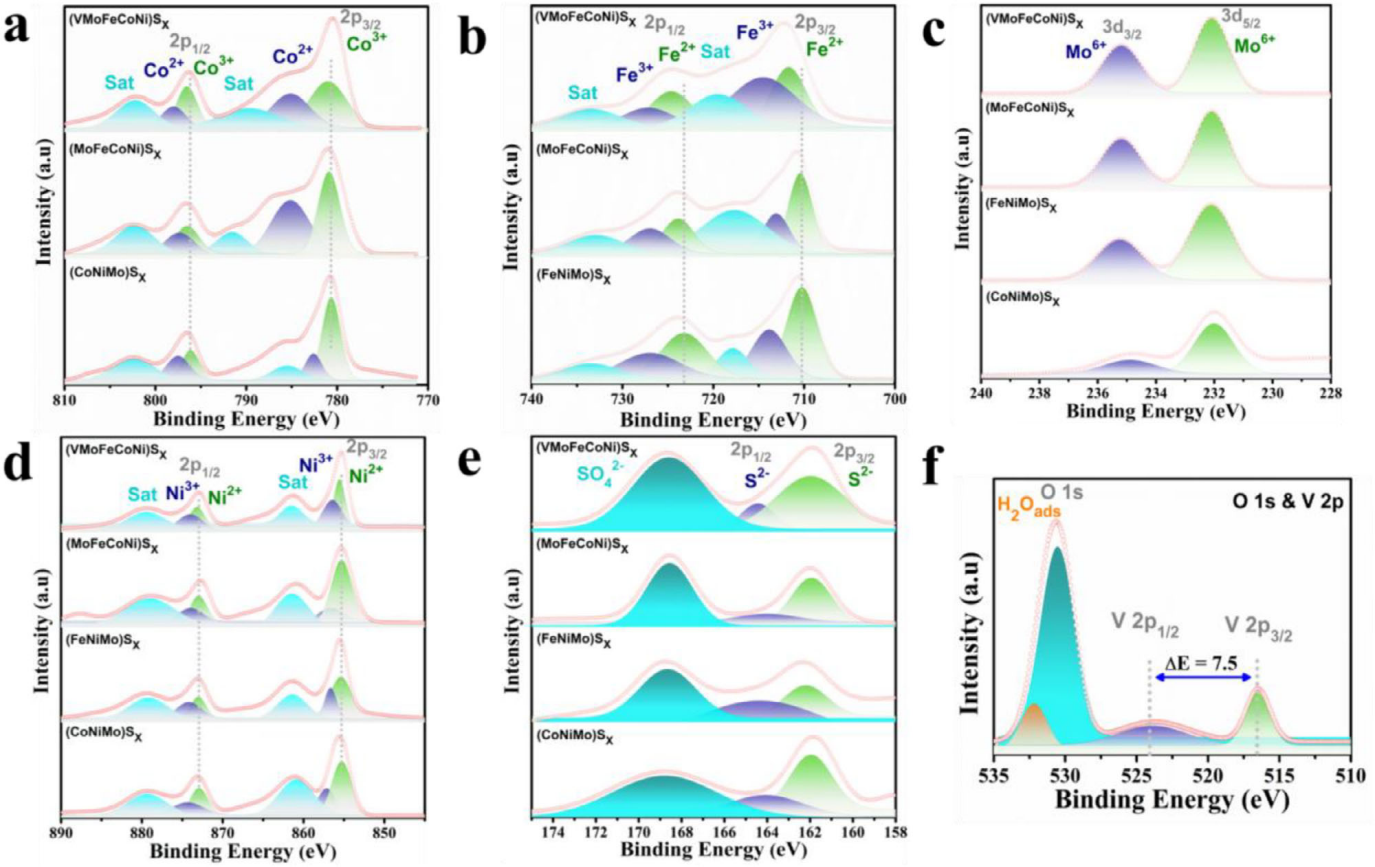
Surface composition and oxidation state analysis. High resolution XPS spectra of (a) Co 2p, (b) Fe 2p, (c) Mo 3d, (d) Ni 2p, (e) S 2p, (f) O 1s &s V 2p.

X‐ray photoelectron spectroscopy (XPS) was utilized to characterize the surface chemical states of the constituent elements in the as‐prepared HEMC sample. The survey spectra, presented in Figure S11, reveal distinct peaks corresponding to vanadium, molybdenum, iron, cobalt, nickel, and sulfur, aligning closely with the elemental composition determined by energy‐dispersive X‐ray spectroscopy (EDS). High‐resolution XPS spectra were acquired to probe the oxidation states of these elements. The Co 2p spectrum of the HEMC (Figure 3a) exhibits two spin‐orbit doublets and two satellite peaks, with binding energies at 780.9 eV and 796.5 eV assigned to Co^3+^, and those at 785.1 eV and 798.0 eV attributed to Co^2+^. The satellite peaks are observed at 789.5 eV and 802.1 eV [28]. The Fe 2p spectrum (Figure 3b) displays two spin‐orbit split doublets (2p_3/2_ and 2p_1/2_), deconvoluted into Fe^2+^ (711.7 eV and 724.7 eV) and Fe^3+^ (714.6 eV and 727.7 eV) [22]. The Mo 3d spectrum (Figure 3c) confirms the presence of Mo^6+^ in the HEMC [40]. Similarly, the Ni 2p spectrum (Figure 3d) is resolved into 2p_3/2_ and 2p_1/2_ doublets, with binding energies at 855.3 eV and 856.4 eV corresponding to Ni^2+^, and 872.9 eV and 873.9 eV assigned to Ni^3+^, accompanied by two satellite peaks [41]. The S 2p spectrum, shown in Figure 3e, reveals two distinct peaks at 162.9 eV and 165.4 eV, corresponding to the S^2−^ 2p_3/2_ and 2p_1/2_ states, respectively. An additional peak at 168.5 eV is attributed to surface SO_4_^2−^ species, which interact with multivalent metal cations to enhance oxygen evolution reaction (OER) performance [27]. The O 1s and V 2p spectra are presented in Figure 3f. The O 1s spectrum is resolved into two components: a dominant peak at 530 eV, indicative of lattice oxygen bonded to metal atoms (M–O), and a minor peak at 531.4 eV, associated with surface oxygen species and adsorbed water molecules. The V 2p spectrum displays two prominent peaks at 516.6 eV (2p_3/2_) and 524.1 eV (2p_1/2_), with a spin‐orbit splitting of 7.5 eV, confirming the presence of V^5+^ oxidation state [42]. The addition of vanadium (second high valent atom) induces a notable shift in the XPS peaks. In particular, the core‐level signals for Fe 2p (±0.5 eV), Ni 2p (±0.2 eV), and Co 2p (±0.4 eV) display a positive shift (charge redistribution), which implies a reduction in the electronic charge surrounding the Fe, Ni, and Co sites. This phenomenon serves as compelling support for an interdependent electronic interaction involving the Fe, Co, Ni, Mo, and V constituents. Consequently, these modifications are anticipated to modulate the binding affinities of OER intermediates at the Co, Ni, and Fe active sites [21, 43].

### 2.3 Electrochemical Activity

**FIGURE 4:**
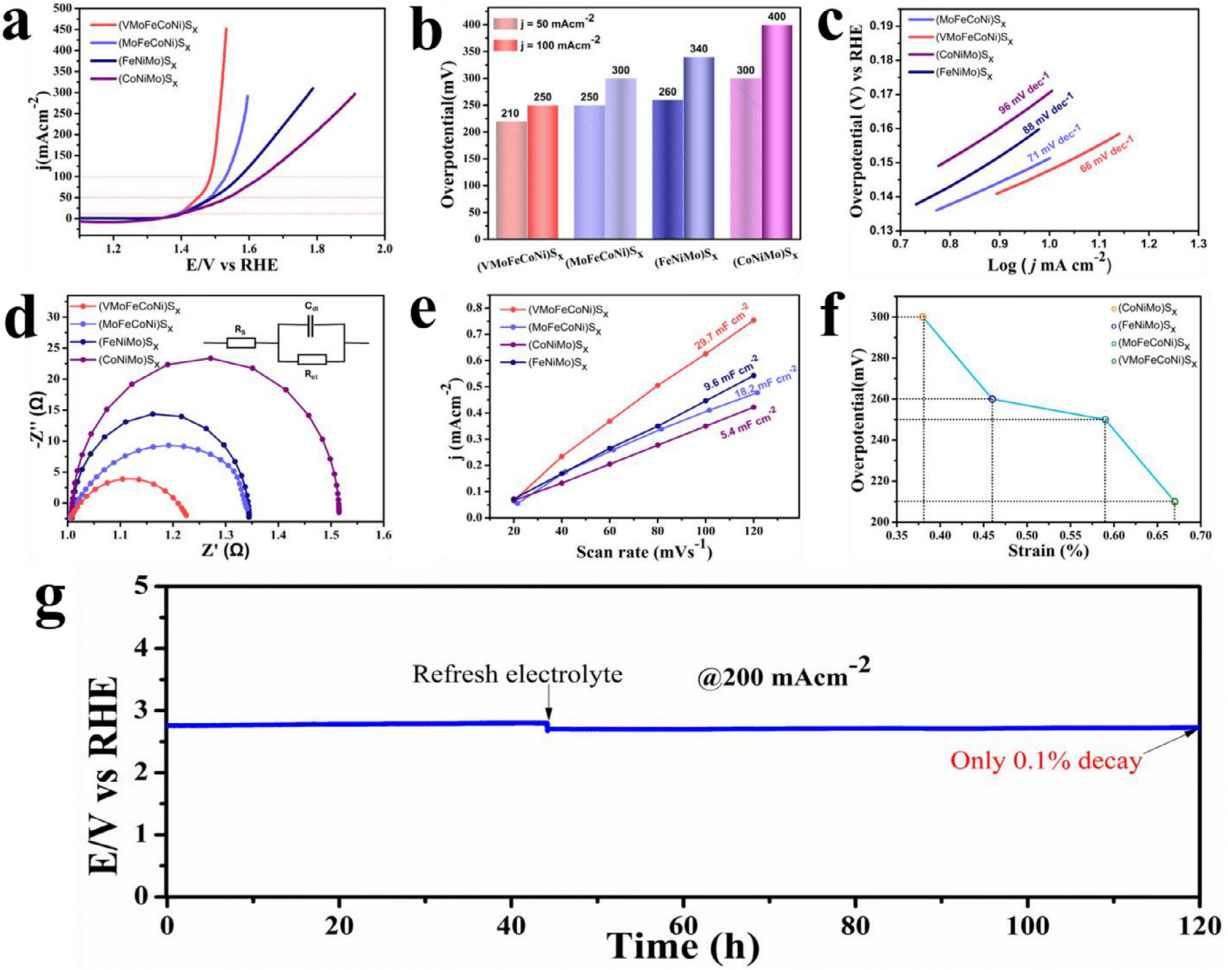
Electrocatalytic performance in alkaline medium: (a) The *iR* corrected Linear sweep voltammetry (LSV) curves for OER of (VMoFeCoNi)S_x_/NF, (MoFeCoNi)S_x_/NF, (FeNiMo)S_x_/NF, and (CoNiMo)S_x_/NF; (b) comparison of overpotentials at current densities of 50 mA cm^−2^ and 100 mA cm^−2^; (c) Tafel slopes extracted from LSV data; (d) electrochemical impedance spectroscopy (EIS) Nyquist plots; (e) double‐layer capacitance measurements; (f) Correlation between micro strain and OER overpotential; (g) chronopotentiometric stability assessment of (VMoFeCoNi)S_x_/NF at a constant current density of 200 mA cm^−2^.

The oxygen evolution reaction (OER) performance of the (VMoFeCoNi)S_x_ HEMC was evaluated in a 1.0 m KOH electrolyte using a standard three‐electrode system. For comparison, ternary (CoNiMo)S_x_, (FeNiMo)S_x_, and quaternary (MoFeCoNi)S_x_ metal sulfides were tested as control samples. Polarization curves were acquired at a scan rate of 2 mV s^−1^ to precisely determine the onset potential, referenced to the reversible hydrogen electrode (RHE). Among the evaluated electrocatalysts, (VMoFeCoNi)S_x_ deposited on nickel foam (NF) exhibited higher electrocatalytic activity, achieving a significantly higher current density within a narrow potential window (Figure 4a). To assess OER activity, current densities of 50 mA cm^−2^ and 100 mA cm^−2^ (denoted as η_50_ and η_100_) were selected as benchmarks, as these metrics are less affected by metal oxidation currents near 1.40 V vs. RHE. The (VMoFeCoNi)S_x_ HEMC demonstrated overpotentials of 210 mV and 250 mV at 50 mA cm^−2^ and 100 mA cm^−2^, respectively, outperforming the control samples (MoFeCoNi)S_x_, (FeNiMo)S_x_, and (CoNiMo)S_x_ by 50, 60, and 90 mV, respectively (Figure 4b). To isolate the catalytic contribution of the HEMC, a bare NF substrate without the catalyst was tested, revealing negligible anodic current compared to (VMoFeCoNi)S_x_ and (Figure S12). Additionally, under identical operating conditions, the HEMC also outperforms the benchmark NiFe (oxy)hydroxide (Figure S13). The OER activity exhibited a clear correlation with the number of metallic elements in the M_x_S_y_ structure, with performance increasing with the introduction of high valent metals. The swift surge of current density within a confined potential range signifies accelerated reaction kinetics. This phenomenon is attributed to the incorporation of high‐valent vanadium and molybdenum alongside iron, cobalt, and nickel, which fosters enhanced covalency between the metal d‐orbitals and sulfur 2p orbitals. This increased covalent interaction promotes efficient electron transfer and reduces the energy barrier for oxygen evolution reaction (OER) intermediates, thereby enhancing catalytic performance. Furthermore, the electrocatalytic performance of (VMoFeCoNi)S_x_ underscores the synergistic interactions within the high‐entropy framework, which effectively tune the electronic structure to optimize catalyst‐adsorbate interactions, thereby enhancing OER efficiency. The (VMoFeCoNi)Sx HEMC on NF exhibits intrinsic electrocatalytic activity, positioning it among the top‐performing anodes reported in the literature (Table S3). To assess the kinetic efficiency of the OER, Tafel slope analysis was conducted to elucidate the reaction dynamics. The (VMoFeCoNi)S_x_/NF catalyst demonstrated a Tafel slope of 66 mV dec^−1^, notably lower than those of the control samples: (MoFeCoNi)S_x_ (71 mV dec^−1^), (FeNiMo)S_x_/NF (88 mV dec^−1^), and (CoNiMo)S_x_/NF (96 mV dec^−1^) (Figure 4c). This low Tafel slope suggests that the formation of the ^*^OOH intermediate is the rate‐determining step (RDS). The OER mechanism involves a sequential process: initially, active metal sites (M) coordinate with hydroxide ions (OH^−^) to form a metal‐hydroxide intermediate (M─OH), accompanied by the release of an electron. This intermediate subsequently transforms into a metal‐oxo species (M═O) with the loss of a second electron. The ^*^OOH species is then formed, which, upon interaction with additional hydroxide ions, regenerates the active metal site while liberating water and molecular oxygen [44]. Electrochemical impedance spectroscopy (EIS) was employed to probe the real‐time resistive properties of electrodes during OER processes. The OER kinetics of the (VMoFeCoNi)S_x_ HEMC were evaluated through EIS, utilizing a Nyquist plot fitted to an equivalent circuit model to determine the charge transfer resistance (Rct) (Figure 4d). Among the tested samples, (VMoFeCoNi)S_x_ exhibited the lowest Rct value of 1.22 Ω, underscoring its excellent charge transfer efficiency and enhanced electrocatalytic performance. To elucidate the intrinsic catalytic activity of OER catalysts, the electrochemically active surface area (ECSA) was determined to quantify the density of exposed active sites. The ECSA was derived from the double‐layer capacitance (Cdl), calculated using the methodology outlined in the Experimental Section. The current density difference (Δj) was plotted against scan rate (Figure S14), and the resulting slope was used to compute Cdl. As shown in Figure 4e, the (VMoFeCoNi)S_x_ HEMC exhibited the highest Cdl value of 29.7 mF cm^−2^ among the tested catalysts, indicating an abundance of active sites that enhance OER kinetics compared to other samples. The direct correlation between lattice micro strain (ɛ) and OER overpotential clearly demonstrates the efficacy of strain‐modulated engineering in HEMC (Figure 4f). As quantified by Williamson‐Hall analysis, our HEMC catalyst, compared to control samples, exhibited the highest micro strain (0.67%), simultaneously achieving the lowest overpotential (210 mV@ 50 mA cm^−2^). This inverse relationship strongly suggests that the compositional complexity inherent to high‐entropy systems induces beneficial lattice distortions, effectively tuning the electronic structure of active sites. This compressive strain modifies the interatomic bond lengths, thereby modulating the overlap of metal d‐orbitals (shifting the d band closer to the Fermi level) to optimize the binding energy of OER intermediates, consistent with the Sabatier principle [45, 46]. More specifically, the strain phenomenon increases the charge transfer rate of OH adsorption in the OER. Although high configurational entropy stabilizes the single‐phase solid solution, the catalytic activity correlates quantitatively with lattice microstrain rather than entropy itself, identifying microstrain as the direct physical descriptor governing OER kinetics. Therefore, it is concluded that entropy provides the thermodynamic stability required to host disparate elements within a single lattice, while it is the strain‐induced geometric compression that acts as the primary physical lever, tuning the d‐band center to lower the activation energy barrier for the rate‐determining step. The excellent electrocatalytic performance of the (VMoFeCoNi)S_x_ HEMC is further demonstrated through its mass activity and exchange current density, as illustrated in Figure S15. Among the evaluated catalysts, (VMoFeCoNi)S_x_ exhibits the highest mass activity, achieving a value of 766.0 A g^−1^ at a potential of 1.53 V, significantly surpassing the performance of ternary and quaternary reference samples. Furthermore, the intrinsic electrocatalytic efficacy of the HEMC is demonstrated through its high electrochemically active surface area (ECSA)‐normalized current density (Figure S16) and turnover frequency (Figure S17), which significantly surpass those of the reference samples. The faradaic efficiency (FE) was further assessed by measuring the gaseous products evolved at the anode (O_2_). The gas volumes collected for HEMC closely matched the theoretical values, confirming a high FE of 97% (Figure S18), indicating that the current during the electrochemical process primarily originates from oxygen evolution. This high FE of HEMC demonstrates its selectivity and stability for oxygen production as compared to its counterparts.

The long‐term stability of electrocatalysts is a pivotal factor in assessing their suitability for practical electrochemical applications. As highlighted in prior studies, such as those by Markovic et al., supporting materials in alkaline media often undergo oxidation at elevated anodic potentials, forming insulating oxide layers that impair electron transfer and compromise catalytic efficiency at the electrode‐electrolyte interface [47]. This oxidative process alters the catalyst's electronic structure, reducing the availability of active sites and hindering interactions with reactant species, ultimately leading to deactivation. To achieve robust performance in harsh alkaline environments, strong electronic coupling and a mechanically stable interface between the catalyst and NF substrate are essential. The durability of the (VMoFeCoNi)S_x_/NF HEMC was evaluated via chronopotentiometry at a constant current density of 200 mA cm^−2^ over 120 h (Figure 4g), significantly outperforming the reference samples (Figure S19). The catalyst exhibited good stability, showing no significant degradation in performance (Figure S20). Specifically, after 120 h at 200 mA cm^−2^, the overpotential at 10 mA cm^−2^ increased by only Δη_10_ = 6 mV, confirming operational stability. This remarkable durability is attributed to several key factors. Primarily, the formation of a stable passivation layer on the catalyst surface serves as a protective barrier, mitigating further oxidative reactions and stabilizing the electrode potential in alkaline conditions. This passivation process, which evolves over several hours to days, optimizes surface chemistry and enhances reaction kinetics, ensuring sustained catalytic activity. Additionally, the hierarchical architecture of the catalyst, characterized by short diffusion pathways, minimizes bubble entrapment during OER, facilitating efficient mass transport and ion diffusion. Notably, the synergistic interplay of alloying elements (Fe, Co, Ni) in the HEMC, often referred to as the cocktail effect, enables rapid formation of a protective passivation layer, effectively preventing further oxidation [48]. Furthermore, the inherent high‐entropy structure of (VMoFeCoNi)S_x_ lowers the system's free energy, enhancing thermodynamic stability and contributing to its robust performance under prolonged operational conditions. Overall, the durability of the HEMC catalyst is attributed to its entropy‐driven phase stabilization and strong interfacial bonding with the nickel foam (NF) substrate, ensuring sustained performance under harsh electrochemical conditions.

### 2.4 Post OER Structural Validation

The excellent OER performance and durability of the (VMoFeCoNi)S_x_ HEMC underscores its excellent electronic and chemical stability under alkaline conditions. To further investigate its post‐OER integrity, SEM analysis was performed, revealing minor macroscopic surface cracks but preservation of the characteristic nanoflake morphology and elemental retention (Figures S21 and S22). Additionally, SEM elemental mapping and EDS spectra analysis exhibited the homogeneous elemental distribution with comparable atomic percentages as that of the pristine sample (Table S4). This structural robustness highlights the outstanding corrosion resistance of the HEMC supported on nickel foam (NF) in 1.0 m KOH electrolyte during the OER stability test. The retention of the nanoflake architecture is crucial, as it maintains a highly electrochemically active surface area and ensures an abundance of catalytic sites, both of which are essential for maximizing water electrolysis efficiency. The post‐chronopotentiometry XRD patterns showed no significant changes (Figure S23), confirming that the HEMC maintained its pristine crystallographic structure, further validating its stability for long‐term electrocatalytic applications. These results underscore that the electrocatalyst manifests not only good electrocatalytic efficacy but also outstanding resilience to corrosive degradation and structural robustness. High‐resolution XPS spectra of the HEMC constituents were acquired after 120 h of OER cycling at 200 mA cm^−2^, to elucidate surface chemical evolution during catalysis as depicted in Figure S24. A comparison of the pre‐ and post‐OER spectra reveals significant transformation in the binding energies and relative percentages of the constituent species, as summarized in Table S5. The Fe 2p, Co 2p, and Ni 2p core‐level spectra reveal systematic shifts toward higher binding energies (+0.6, +0.5, +0.3, respectively), confirming the charge redistribution and partial oxidation to (oxy) hydroxide species during OER. This is consistent with the formation of higher‐valence metal oxide species at the catalyst surface with a marked increase in their relative proportions (Figure S25). Specifically, the post‐OER Co 2p_3/2_, Fe 2p_3/2_, Ni 2p_3/2_, and Mo 3d_5/2_ spectra reveal an increase in the relative percentages along with a binding energy shift signifying a progressive oxidation that enhances electronic interactions within the lattice. Such oxidative reconfiguration underscores a resilient catalytic scaffold capable of sustaining efficient electron transport and active‐site turnover, ultimately accelerating the OER process. Moreover, the V 2p signals are effectively suppressed, arising from the dissolution and leaching of vanadium, which promotes the deposition of a metal oxyhydroxide (MOOH) passivation layer. Additionally, a distinctive O 1s peak emerges at 531.0 eV, attributable to M─OH moieties, thereby substantiating the oxidative reconstruction of the sulfide scaffold into an (oxy)hydroxide phase. Notably, the remaining sulfur mainly exists as sulfate (SO_4_^2−^). Its presence alongside the (oxy)hydroxide phases likely boosts OER activity through synergistic electronic effects. Mechanistically, although the surface undergoes oxidative reconstruction during catalysis, the retention of the bulk pyrite phase, as evidenced by XRD analysis (Figure S22), confirms the formation of a stable core‐shell architecture. This initial strain‐engineered sulfide core plays a critical role by acting as a highly conductive scaffold that facilitates rapid electron transport to the surface‐active layer, effectively overcoming the intrinsic low conductivity of pure oxyhydroxides. Furthermore, the substantial leaching of vanadium observed in the post‐OER XPS (Table S5) functions as an in‐situ etching‐induced vacancy generation process. Since the initial high‐entropy configuration ensured an atomic‐scale distribution of vanadium, its selective dissolution creates a homogeneous, high‐density array of cation vacancies and unsaturated coordination sites within the active shell. Consequently, the superior OER performance arises from a synergistic coupling where the preserved high‐entropy sulfide core maintains lattice strain and conductivity, while the reconstructed shell provides abundant defect‐rich active sites derived from the sacrificial leaching of the lattice‐straining vanadium. While operando characterization would provide direct real‐time observation of dynamic reconstruction, the convergent agreement of retained bulk strain (XRD), surface oxidation behavior (XPS), and strain–activity scaling establishes a causally consistent mechanistic framework sufficient for interpretation at this stage. Importantly, the enhanced catalytic activity observed in this work cannot be reproduced by directly synthesizing compositionally similar multimetal (oxy)hydroxides. In the absence of the strain‐preserved high‐entropy sulfide parent lattice, direct hydroxide synthesis inevitably leads to lattice relaxation and partial cation segregation, which suppresses both homogeneous vacancy formation and interfacial strain coupling. In contrast, reconstruction from the entropy‐stabilized sulfide uniquely preserves bulk compressive strain and enforces coherent strain transfer across the sulfide/oxyhydroxide interface, resulting in an electronically modulated active shell that cannot be accessed through direct hydroxide synthesis routes. Overall, these results strongly confirm the structural changes in HEMC during OER, consistent with prior studies. To rationalize the observed enhancement in catalytic performance, we distinguish the contribution of geometric factors arising from the nanoflake morphology from intrinsic electronic effects governed by lattice strain. The HEMC exhibits a double‐layer capacitance (*C_dl_*) of 29.7 mF cm^−2^, approximately double that of the ternary controls, demonstrating that the high‐entropy synthesis promotes a highly porous, hierarchical nanoflake architecture that increases electrolyte contact. However, geometric area alone cannot account for the superior performance. As evident from the ECSA‐normalized polarization curves (Figure S16) and Turnover Frequency analysis (Figure S17), the HEMC exhibits superior intrinsic kinetics per active site. This observation highlights a cooperative effect in which the nanoflake architecture maximizes the availability of active sites, while lattice strain fine‐tunes their electronic structure to enhance intrinsic catalytic efficiency. Notably, in light of the complexities regarding surface reconstruction and random atomic distribution in high‐entropy alloys, we deliberately focused on experimentally verified descriptors rather than idealized simulations to elucidate the catalytic mechanism. The strong empirical correlation observed between micro‐strain and catalytic activity serves as a definitive indicator of electronic structure modulation. This allows us to rigorously attribute the performance enhancement to strain‐induced optimization of the d‐band center, validating the ‘strain‐modulated engineering’ concept through physical evidence rather than theoretical concept, consistent with established trends in strain engineering [49].

## 3 Conclusions

This study presents a sophisticated strain‐engineering multilayered electrode design featuring a nanoflake morphology, offering a robust platform for efficient oxygen evolution reaction (OER) electrocatalysis. The precise manipulation of electronic structure and nanoscale architecture represents a breakthrough approach to overcoming the activity‐stability dilemma in OER catalysts. The ultrathin nanosheet architecture provides a high surface area, significantly enhancing catalyst exposure and maximizing the availability of active sites for water oxidation. The incorporation of high‐valent metals into the HEMC matrix strengthens the interaction between the catalyst and substrate, while markedly lowering charge transfer resistance, thereby improving electron transport efficiency. Additionally, the integration of multiple metallic elements triggers a 0.67% strain, thereby modulating the coordination environment within the HEMC. As a result, it optimizes the electronic structure to increase the density of catalytic sites and boost stability under harsh alkaline conditions. This work not only establishes nanoflake‐based HEMCs as highly effective electrodes for low‐voltage OER systems but also provides valuable insights into the unique catalytic mechanisms of high‐entropy materials, paving the way for their application in advanced energy conversion technologies.

## 4 Experimental Section

### 4.1 Preparation of HEMC

The (VMoFeCoNi)S_x_ high‐entropy multi‐metallic catalyst (HEMC) was synthesized through an efficient two‐step solvothermal reduction process. In the initial step, a homogeneous solution was prepared by dissolving 0.14 mmol of (NH_4_)_6_Mo_7_O_24_·4H_2_O and 13 mmol of CO(NH_2_)_2_ in 25 mL of deionized water, facilitated by ultrasonication. To this solution, 1.2 mmol each of NH_4_VO_3_, Fe (NO_3_)_3_·9H_2_O, Co (NO_3_)2·6H_2_O, and Ni (NO_3_)_2_·6H_2_O, along with 2 mmol of CH_4_N_2_S and 0.2 g of polyvinylpyrrolidone (PVP) as a stabilizer, were added and ultrasonicated for 1 h to ensure uniformity. The resulting mixture was transferred to a 100 mL Teflon‐lined stainless‐steel autoclave, sealed, and heated to 100°C for 8 h, followed by cooling to ambient temperature. The product was washed three times with a deionized water/ethanol mixture and dried at 70°C for 12 h. In the second step, the dried material was placed in a 50 mL Teflon‐lined autoclave containing 0.7 g of sodium hydroxide, 0.1 g of sodium borohydride, and 25 mL of ethylene glycol, and maintained at 190°C for 10 h. The final (VMoFeCoNi)S_x_ HEMC was thoroughly washed with deionized water/ethanol and dried under vacuum.

Reference samples, including (MoFeCoNi)S_x_, (FeNiMo)S_x_, and (CoNiMo)S_x_, were synthesized using an analogous procedure, omitting vanadium or other metals as required to maintain compositional consistency for comparative studies.

### 4.2 Material Characterizations (Structural and Chemical Evaluation)

A Rigaku Ultima III X‐ray diffractometer equipped with a Cu‐Kα source (λ = 1.5418 Å) was employed to investigate the phase structure of the catalysts. The lattice strain (ɛ) of all the analysed samples was quantified from PXRD data using the following Williamson‐Hall equation.

$$\beta_{\mathrm{hkl}}.\cos\theta_{\mathrm{hkl}}=K\lambda /d+4\varepsilon .\sin\theta_{\mathrm{hkl}}$$

where (K) is the Scherrer constant (0.9)

λ = Cu‐Kα radiation source (1.5418 Å)

D = Crystallite size

β_hkl_ = Full Width at Half Maximum (FWHM) in radians, obtained by measuring the width of the diffraction peak at half of its maximum intensity

Ѳ = Bragg angle, determined from the corresponding PXRD pattern

According to the linear relationship between β_hkl_.cosθ_hkl_ and 4ɛ.sinθ_hkl_, the crystallite size and the micro lattice strain (ɛ) were determined from the intercept and slope of the linear fit, respectively.

Raman spectra were acquired using micro‐Raman microscopy (Renishaw, India) with a 532 nm laser. The surface morphology of the materials was examined using a field‐emission scanning electron microscope (SEM, JEOL JSM‐7401F) and a transmission electron microscope (FEI, Talos F200x) equipped with a STEM‐EDS detector was accomplished to explore the in‐depth microstructures of HEMC. X‐ray photoelectron spectroscopy (XPS, Thermo VG, Microtech ESCA) was utilized to analyse the surface elemental composition and oxidation states.

### 4.3 Electrochemical Measurements

Electrochemical experiments were conducted using a Metrohm Autolab PGSTAT204 workstation at a low scan rate of 2 mV s^−1^, employing a conventional three‐electrode configuration in a 1.0 m KOH electrolyte at ambient temperature. The working electrode consisted of nickel foam, with Hg/HgO serving as the reference electrode and a graphite rod as the counter electrode. To prepare the working electrode, a catalyst ink was prepared by dispersing 6 mg of the synthesized catalyst in a mixture of 400 µL deionized water, 700 µL absolute ethanol, and 20 µL of 5.0 wt% Nafion, homogenized via ultrasonication for 60 min. The ink (35 µL) was applied onto a nickel foam substrate (0.5 cm × 1 cm) using a drop‐casting technique and dried at 60°C for 12 h before testing. To evaluate the electrochemically active surface area (ECSA), cyclic voltammetry (CV) was performed at scan rates of 20 mV s^−^^1^, 40 mV s^−^^1^, 60 mV s^−^^1^, 80 mV s^−^^1^, and 120 mV s^−^^1^ in the non‐Faradaic region (0–0.8 V vs. RHE). Linear sweep voltammetry (LSV) polarization curves were recorded at 2 mV s^−1^ with iR compensation applied using the equation E_corrected_ = E − iRs, where Rs represents the solution resistance determined from EIS in 1.0 m KOH, and i denotes the working electrode current. All potentials were referenced to the reversible hydrogen electrode (RHE), with overpotentials calculated as η = E_RHE_ − 1.23 V. The double‐layer capacitance (Cdl) was determined by plotting the charging current (*Ic*, mA cm^−2^) against scan rate (V, mV s^−1^) in the non‐Faradaic region, using the equation Cdl = *Ic*/V. The Cdl values were normalized using a specific capacitance of 0.35 mF cm^−2^ to estimate ECSA, and ECSA‐normalized current densities were calculated as j_ECSA‐normalized_ = j/ECSA. Charge transfer resistance was assessed via electrochemical impedance spectroscopy (EIS) in the frequency range of 100 kHz to 0.1 Hz at a potential of 1.50 V vs. RHE, with an amplitude of 5 mV. Long‐term stability was evaluated chronopotentiometrically (V‐t curves) using a platinum mesh counter electrode, substituted for the graphite rod to ensure stability at high current densities. Additional electrochemical parameters, including mass activity and exchange current density, were derived using standard equations detailed in the Supporting Information. Post‐stability structural and morphological analyses were conducted using powder X‐ray diffraction (PXRD) and scanning electron microscopy (SEM), following established protocols.

## Conflicts of Interest

The authors declare no conflict of interest.

## Supporting information




**Supporting File**: smll73201‐sup‐0001‐SuppMat.docx.

## Data Availability

The data that support the findings of this study are available from the corresponding author upon reasonable request.

## References

[1] T. B. T. Huynh , Q. T. Tran , N. Q. Diep , et al., “Alloying‐Induced Crystal‐Phase Transition in In_x_Ga_y_Se_z_ Alloys Grown on C‐Sapphire Substrates by Molecular Beam Epitaxy: Implication for Next‐Generation Optoelectronics,” Crystal Growth & Design 25, no. 12 (2025): 4159–4168, 10.1021/acs.cgd.4c01750.

[2] J. W. Turnley and R. Agrawal , “Solution Processed Metal Chalcogenide Semiconductors for Inorganic Thin Film Photovoltaics,” Chemical Communications 60, no. 40 (2024): 5245–5269, 10.1039/D4CC01057D. 38683572

[3] M. A. Buckingham , J. J. Shea , K. Z. Quan , et al., “Using High Pressure to Investigate the Stability of a High Entropy Wurtzite Structured (MnFeCuAgZnCd)S,” Communications Chemistry 8, no. 1 (2025): 65, 10.1038/s42004-025-01463-9. 40044902 PMC11882918

[4] E. J. Miller , K. P. Robinson , E. Lara , et al., “Atomically Engineered Active Sites in Metal Trichalcogenides via Dual Sublattice Modulation for Electrocatalysis Applications,” ACS Applied Nano Materials 8, no. 32 (2025): 15869–15882, 10.1021/acsanm.5c02546.

[5] M. Zubair , D. Lee , and D. J. Kang , “Harnessing the Power of 2D Materials for Flexible Energy Harvesting Applications,” Carbon Energy 7 (2025): 70083, 10.1002/cey2.70083.

[6] L. Wu , X. Shen , Z. Ji , et al., “Facile Synthesis of Medium‐Entropy Metal Sulfides as High‐Efficiency Electrocatalysts Toward Oxygen Evolution Reaction,” Advanced Functional Materials 33, no. 3 (2023): 2208170, 10.1002/adfm.202208170.

[7] K. D. Ramadhass and C. C. Lin , “3D Nanoflowers of Binary Metal‐Selenide for Improved Electrochemical Sensing and High‐Energy‐Density Energy Storage,” Small 21 (2025): 2505860, 10.1002/smll.202505860. 40641292 PMC12444850

[8] S. Singh , A. Irshad , G. D. De la Cruz , et al., “Bifunctional Noble Metal‐Free Ternary Chalcogenide Electrocatalysts for Overall Water Splitting,” Chemistry of Materials 37, no. 3 (2025): 823–832, 10.1021/acs.chemmater.4c01684.

[9] S. Chen , B. Zu , Q. Jin , X. Wu , Z. Xu , and L. Wu , “General Synthesis of Wurtzite Cu‐Based Quaternary Selenide Nanocrystals via the Colloidal Method,” ACS Applied Materials & Interfaces 17, no. 16 (2025): 24382–24389, 10.1021/acsami.5c04244. 40229196

[10] S. Mete , M. S. Sengar , M. Dhayal , V. Kumar , and S. K. Singh , “Lattice Strain‐Induced Electronic Effects on a Heteroatom‐Doped Nickel Alloy Catalyst for Electrochemical Water Splitting,” Journal of Materials Chemistry A 12, no. 46 (2024): 32371–32384, 10.1039/D4TA05604C.

[11] B. Wang , W. Liu , Y. Leng , et al., “Strain Engineering of High‐Entropy Alloy Catalysts for Electrocatalytic Water Splitting,” Iscience 26, no. 4 (2023): 106326. 36950114 10.1016/j.isci.2023.106326 PMC10025961

[12] L. Yao , F. Zhang , S. Yang , et al., “Sub‐2 nm IrRuNiMoCo High‐Entropy Alloy with Iridium‐Rich Medium‐Entropy Oxide Shell to Boost Acidic Oxygen Evolution,” Advanced Materials 36, no. 25 (2024): 2314049, 10.1002/adma.202314049. 38516927

[13] S. S. Almishal , M. Furst , Y. Tan , et al., “Thermodynamics‐Inspired High‐Entropy Oxide Synthesis,” Nature Communications 16, no. 1 (2025): 8211, 10.1038/s41467-025-63567-z. PMC12405472 40897701

[14] Y. Ye , Z. Gu , J. Geng , et al., “Advanced High‐Entropy Halide Solid Electrolytes Enabling High‐Voltage, Long‐Cycling All‐Solid‐State Batteries,” Nano Letters 25, no. 10 (2025): 3747–3755, 10.1021/acs.nanolett.4c05460. 40015691

[15] G. R. Dey , S. S. Soliman , and R. E. Schaak , “Three Complementary Strategies for Synthesizing Colloidal Nanoparticles of High Entropy Transition Metal Ditellurides,” Inorganic Chemistry 64, no. 26 (2025): 13446–13455, 10.1021/acs.inorgchem.5c01971. 40552770

[16] F. Zhang , T. Gao , Y. Zhang , et al., “High‐Entropy Metal Sulfide Nanocrystal Libraries for Highly Reversible Sodium Storage,” Advanced Materials 37 (2025): 2418890, 10.1002/adma.202418890. 40091399

[17] M. Chandran , P. Dutta , P. Singh , A. K. Singh , and B. L. Prasad , “Design and Synthesis of PtPdNiCoMn High‐Entropy Alloy Electrocatalyst for Enhanced Alkaline Hydrogen Evolution Reaction: A Theoretically Supported Predictive Design Approach,” Advanced Functional Materials 35, no. 17 (2025): 2418644, 10.1002/adfm.202418644.

[18] R. Mohili , N. Hemanth , H. Jin , K. Lee , and N. Chaudhari , “Emerging High Entropy Metal Sulphides and Phosphides for Electrochemical Water Splitting,” Journal of Materials Chemistry A 11, no. 20 (2023): 10463–10472, 10.1039/D2TA10081A.

[19] A. Abdelhafiz , B. Wang , A. R. Harutyunyan , and J. Li , “Carbothermal Shock Synthesis of High Entropy Oxide Catalysts: Dynamic Structural and Chemical Reconstruction Boosting the Catalytic Activity and Stability Toward Oxygen Evolution Reaction,” Advanced Energy Materials 12 (2022): 2200742, 10.1002/aenm.202200742.

[20] D. Wang , Y. Zhang , C. Zhao , et al., “Electronic Structure Modulation Coupled with Vacancy Defect Engineering in FeCoNiCrV High‐Entropy Alloy Electrocatalyst for Enhanced Oxygen Evolution Reaction,” ACS Applied Materials & Interfaces 17, no. 32 (2025): 45687–45695, 10.1021/acsami.5c08254. 40736332

[21] Y. Mei , Y. Feng , C. Zhang , Y. Zhang , Q. Qi , and J. Hu , “High‐Entropy Alloy with Mo‐Coordination as Efficient Electrocatalyst for Oxygen Evolution Reaction,” ACS Catalysis 12, no. 17 (2022): 10808–10817, 10.1021/acscatal.2c02604.

[22] A. Sivanantham , H. Lee , S. W. Hwang , et al., “Complementary Functions of Vanadium in Boosting Electrocatalytic Activity of CuCoNiFeMn High‐Entropy Alloy for Water Splitting,” Advanced Functional Materials 33, no. 34 (2023): 2301153, 10.1002/adfm.202301153.

[23] S. Gao , H. You , W. Xia , et al., “Vanadium Doping for Enhanced Electrochemical Performance of the High‐Voltage LiCu_0.5_Mn_1.5_O_4_ Cathode,” Industrial & Engineering Chemistry Research 64, no. 22 (2025): 10864–10872, 10.1021/acs.iecr.5c00828.

[24] S. N. Upadhyay , V. Kumar , N. Sharma , and S. Pakhira , “Enhanced Catalytic Performance of Vanadium‐Doped MoS_2_ as a Multifunctional Electrocatalyst Toward ORR, OER, and HER Applications,” ACS Applied Energy Materials 8, no. 13 (2025): 8937–8949, 10.1021/acsaem.5c00257.

[25] M. Kwon , J. S. Ha , D. H. Lee , et al., “Boosting the Performance of Alkaline Anion Exchange Membrane Water Electrolyzer with Vanadium‐Doped NiFe_2_O_4_ ,” Small 21, no. 7 (2025): 2410006, 10.1002/smll.202410006. 39777981

[26] M. Cui , C. Yang , B. Li , et al., “High‐Entropy Metal Sulfide Nanoparticles Promise High‐Performance Oxygen Evolution Reaction,” Advanced Energy Materials 11, no. 3 (2021): 2002887, 10.1002/aenm.202002887.

[27] H. Cai , S. He , H. Yang , et al., “Highly Exposed Ultra‐Small High‐Entropy Sulfides with d‐p Orbital Hybridization for Efficient Oxygen Evolution,” Advanced Materials 37, no. 33 (2025): 2508610, 10.1002/adma.202508610. 40459467

[28] W. Wang , Z. Yu , L. Yue , et al., “Regulating Electronic Structure and Coordination Environment of Transition Metal Selenides Through the High‐Entropy Strategy for Expedited Lithium–Sulfur Chemistry,” ACS Nano 19, no. 30 (2025): 27440–27454, 10.1021/acsnano.5c05720. 40690885

[29] Q. Li , Y. Zhang , X. Guo , et al., “Nucleation and Growth Mechanisms of Micro/Nano Structural Manganese‐Trimesic Acid Coordinations for Aqueous Zinc‐Ion Batteries,” Angewandte Chemie International Edition 64, no. 31 (2025): 202509741, 10.1002/anie.202509741. 40406804

[30] M. Chandran , S. Sahoo , A. K. Singh , and B. L. Prasad , “Synthesis Framework for Designing PtPdCoNiMn High‐Entropy Alloy: A Stable Electrocatalyst for Enhanced Alkaline Hydrogen Evolution Reaction,” Small 21, no. 1 (2025): 2408317, 10.1002/smll.202408317. 39548920

[31] L. M. Lyu , Y. C. Chang , H. J. Li , et al., “Turning the Surface Electronic Effect Over Core‐Shell CoS_2_─Fe_x_Co_1‐x_S_2_ Nanooctahedra Toward Electrochemical Water Splitting in the Alkaline Medium,” Advanced Science 12, no. 3 (2025): 2411622, 10.1002/advs.202411622. 39605090 PMC11744711

[32] J. Yi , Q. Deng , H. Cheng , D. Zhu , K. Zhang , and Y. Yang , “Unique Hierarchically Structured High‐Entropy Alloys with Multiple Adsorption Sites for Rechargeable Li–CO_2_ Batteries with High Capacity,” Small 20, no. 34 (2024): 2401146, 10.1002/smll.202401146. 38618939

[33] B. Jiang , Y. Yu , J. Cui , et al., “High‐Entropy‐Stabilized Chalcogenides with High Thermoelectric Performance,” Science 371, no. 6531 (2021): 830–834, 10.1126/science.abe1292. 33602853

[34] H. Kang , X. Yang , X. Sun , C. Wang , and P. Xiao , “Microstructure and Properties of CuCrNixTiZr High Entropy Alloys: Experiments and First Principles Calculations,” Journal of Alloys and Compounds 1010 (2025): 177873, 10.1016/j.jallcom.2024.177873.

[35] P. Thirathipviwat , G. Song , J. Bednarcik , U. Kühn , T. Gemming , and K. Nielsch , “Compositional Complexity Dependence of Dislocation Density and Mechanical Properties in High Entropy Alloy Systems,” Progress in Natural Science: Materials International 30, no. 4 (2020): 545–551, 10.1016/j.pnsc.2020.07.002.

[36] N. J. Usharani , R. Shringi , H. Sanghavi , S. Subramanian , and S. Bhattacharya , “Role of Size, Alio‐/Multi‐Valency and Non‐Stoichiometry in the Synthesis of Phase‐Pure High Entropy Oxide (Co,Cu,Mg,Na,Ni,Zn)O,” Dalton Transactions 49, no. 21 (2020): 7123–7132, 10.1039/D0DT00958J. 32406896

[37] P. Wang , G. Wang , K. Chen , et al., “High‐Power Hybrid Alkali‐Acid Fuel Cell for Synchronous Glycerol Valorization Implemented by High‐Entropy Sulfide Electrocatalyst,” Nano Energy 118 (2023): 108992, 10.1016/j.nanoen.2023.108992.

[38] A. Salian , L. L. Praveen , and S. Mandal , “Role of Mg–O on Phase Stabilization in Solution Combustion Processed Rocksalt Structured High Entropy Oxide (CoCuMgZnNi)O with High Dielectric Performance,” Ceramics International 49, no. 19 (2023): 31131–31143, 10.1016/j.ceramint.2023.07.058.

[39] R. Wei , X. Bu , W. Gao , et al., “Interfaces,” ACS Applied Materials & Interfaces 11, no. 36 (2019): 33012–33021. 31414595 10.1021/acsami.9b10868

[40] C. Feng , Y. Zhou , Z. Xie , et al., “Vanadium Boosted High‐Entropy Amorphous FeCoNiMoV Oxide for Ampere‐Level Seawater Oxidation,” Chemical Engineering Journal 495 (2024): 153408, 10.1016/j.cej.2024.153408.

[41] T. E. Meyer , C. C. Peng , C.‐Y. Lin , et al., “Colloidal Synthesis of Thiospinel High‐Entropy Sulfide Star‐Like Nanocrystals with High Cycling Stability for the Oxygen Evolution Reaction,” Nano Letters 25, no. 11 (2025): 4234–4241, 10.1021/acs.nanolett.4c05699. 40062837

[42] H. Cho , Y. Kim , J. K. Kim , M. H. Kim , and H. K. Yu , “High‐Entropy Synthesis of (Cr_0. 7_Co_0. 2_Rh_0. 1_) VO_4_ Nanospheres with Improved Visible‐Light Photoresponse,” Crystal Growth & Design 25, no. 16 (2025): 6954–6962.

[43] Y. Wan , W. Wei , S. Ding , L. Wu , and X. Yuan , “Stabilizing Polyoxometalate for Enhanced OER Performance Using a Porous Manganese Oxide Support,” Small 20, no. 46 (2024): 2404689.

[44] M. Zubair , L. Shen , T. Hyeong Lee , Y. Qian , and D. Joon Kang , “Stabilizing Polyoxometalate for Enhanced OER Performance Using a Porous Manganese Oxide Support,” ChemSusChem 18, no. 9 (2025): 202402294. 10.1002/cssc.202402294 39726113

[45] L. Jiao , E. Liu , S. Hwang , S. Mukerjee , and Q. Jia , “Compressive Strain Reduces the Hydrogen Evolution and Oxidation Reaction Activity of Platinum in Alkaline Solution,” ACS Catalysis 11, no. 13 (2021): 8165–8173.

[46] J. Wang , J. Zhang , H. Yu , L. Chen , H. Jiang , and C. Li , “Strain Engineering of High‐Entropy Oxides Enriches Highly Active Lattice Oxygen for Electrocatalytic Water Oxidation,” ACS Materials Letters 6, no. 5 (2024): 1739–1745.

[47] S. H. Chang , N. Danilovic , K.‐C. Chang , et al., “Functional Links Between Stability and Reactivity of Strontium Ruthenate Single Crystals During Oxygen Evolution,” Nature Communications 5, no. 1 (2014): 4191. 10.1038/ncomms5191 24939393

[48] F. Li , Y. Ma , H. Wu , et al., “Sub‐3‐nm High‐Entropy Metal Sulfide Nanoparticles with Synergistic Effects as Promising Electrocatalysts for Enhanced Oxygen Evolution Reaction,” The Journal of Physical Chemistry C 126, no. 43 (2022): 18323–18332.

[49] B. Guo , Z. Zhou , W. Sun , and X. Hu , “High Entropy Alloys for Advanced Electrocatalysis with Computational Insights and Multidisciplinary Design Strategies,” iScience 28, no. 10 (2025): 113577. 41079622 10.1016/j.isci.2025.113577 PMC12513287

