# Supplementary material for: Strain‐Modulated Engineering of High‐Entropy Vanadium‐Based Chalcogenide for Sustainable Water Oxidation
Source: Small. 2026 Mar 22;22(24):e73201. doi: 10.1002/smll.73201 (PMC13114511; doi:10.1002/smll.73201)
Supplement: Supplementary file 1 — Supporting File: smll73201‐sup‐0001‐SuppMat.docx. [file SMLL-22-e73201-s001.docx]

**Strain-Modulated Engineering of High-Entropy Vanadium-Based Chalcogenide for Sustainable Water Oxidation**

Muhammad Zubair^1^, Yongteng Qian^2^*, Kyung-Ho park^3^, Dae Joon Kang^1^*

*^1^Department of Physics, Sungkyunkwan University, 2066 Seobu-ro, Jangan-gu, Suwon, Gyeonggi-do, 16419 Republic of Korea*

*^2^Pharmaceutical Engineering College, Jinhua University of Vocational Technology, Jinhua, Zhejiang Province, 321007, P.R. China*

*^3^Korea Advanced Nano Fab Center (KANC), Suwon 16229, Republic of Korea*

[*20221132@jhc.edu.cn](mailto:*20221132@jhc.edu.cn), [*djkang@skku.edu](mailto:*djkang@skku.edu)

**Calculation of dislocation density and average crystallite size from the XRD data**

Dislocation density (δ) = 1/D^2^

The average crystallite size of the synthesized materials was calculated using the Debye-Scherrer equation applied to the X-ray diffraction (XRD) patterns.

D = Kλ/βcosѲ

where (K) is the Scherrer constant (0.9)

λ = Cu-Kα radiation source (1.5418 Å)

β = Full Width at Half Maximum (FWHM) in radians, obtained by measuring the width of the diffraction peak at half of its maximum intensity

Ѳ = Bragg angle, determined from the PXRD pattern

**Electrochemical parameters calculation**

E_RHE_ = E_Hg/HgO_ + 0.098 + 0.059 pH

Where, E_RHE_ represents the reversible hydrogen electrode potential, E_Hg/HgO_ is the experimentally determined potential, 0.098 is the potential of a reference electrode (Hg/HgO), 0.059 is the Nernst constant, and pH is the electrolyte pH (1M KOH = 13.9 pH).

Tafel equation η = b log*j* + a

Where η represents the overpotential, b is the Tafel slope, and *j* is the cathodic current density.

Mass activity MA (A/g) = *j*/m

Where *j* represents the current density and m is the loaded mass.

Exchange current density from EIS i_0_ = RT/nFѲ

Where R demonstrates the general gas constant, T is the reaction temperature, n is the number of electrons transferred during OER (i.e., 4 electrons), and Ѳ is the resistance acquired from EIS data.

**(VMoFeCoNi)S_x_**

$$\frac{8.314 J/K .mol \times298 K}{4\times96485 C/mol \times1.22\Omega\times1 \mathrm{cm}^{2}}$$

= 5.26 mA/cm^2^

**(MoFeCoNi)S_x_**

$$\frac{8.314 J/K .mol \times298 K}{4\times96485 C/mol \times1.33\Omega\times1 \mathrm{cm}^{2}}$$

= 4.82 mA/cm^2^

**(FeNiMo)S_x_**

$$\frac{8.314 J/K .mol \times298 K}{4\times96485 C/mol \times1.34\Omega\times1 \mathrm{cm}^{2}}$$

= 4.79 mA/cm^2^

**(CoNiMo)S_x_**

$$\frac{8.314 J/K .mol \times298 K}{4\times96485 C/mol \times1.51\Omega\times1 \mathrm{cm}^{2}}$$

= 4.25 mA/cm^2^

The turnover frequency (Apparent TOF) was quantified using the following equation, reflecting the intrinsic catalytic activity of the system.

TOF = $\frac{j \times A}{4 \times F \times n}$

Where j represents the current density at a specified overpotential, A denotes the electrode’s geometric surface area, F is the Faraday constant, and n corresponds to the molar quantity of active metal sites on the electrode. In this study, all metal components were considered catalytically active, with the molar quantity (n) determined based on the total mass loading of the catalyst, as detailed in the provided equation.

$\mathcal{n}$ = $\frac{\mathcal{m}}{Mw}$

Where m denotes the loaded mass, Mw represents the molecular weight of the catalysts.

**Faradaic efficiency**

The evolved molecular O_2_ was quantified using a water‐displacement method. Gas volumes were calculated using the ideal gas law, assuming that all electrons passing through the external circuit originated exclusively from the oxygen evolution reaction and were fully accounted for in the measured gas production. The measurements were performed in an airtight electrochemical cell connected to a water‐filled graduated burette. Before chronopotentiometric testing, the system was purged with high‐purity N_2_ for 10 min to eliminate residual oxygen, and the initial water level was recorded. During electrolysis, the progressive displacement of water reflected the accumulation of gaseous products in the burette headspace. The experimentally collected gas volumes were then compared with the theoretical values derived from Faraday’s law to determine the Faradaic efficiency.

Faradaic efficiency = $\frac{V_{experimental}}{V_{theoretical}}$

V Theoretical for O2 = 1/4* Q/F * V m

Where 1 in the above equation means 1 mole of O_2_ per mole of H_2_O, and 4 corresponds to 4 moles of electrons per mole of H_2_O.

**Figure S1**. Williamson-Hall plots for lattice strain calculation of (a) (VMoFeCoNi)S_x_, (b) (MoFeCoNi)S_x_, (c) (FeNiMo)S_x_, and (d) (CoNiMo)S_x_

**Figure S2**. The crystal lattice fringes collected from HEMC different nanoflowers/ nanosheets, showing well-defined crystal structure consistent with the XRD results.

**Figure S3**. HAADF-STEM images and corresponding EDS elemental mapping of HEMC nanoflower/nanosheets, representing the uniform distribution of V, Mo, Fe, Co, Ni, and S within a single-phase matrix without any significant impurities.

**Figure S4**. EDS spectra of the HEMC validate the precise stoichiometric composition and accurate atomic ratios of its constituent elements, including vanadium, molybdenum, iron, cobalt, nickel, and sulfur.

**Figure S5**. (a-c) SEM images of (MoFeCoNi)S_x_ at different magnifications.

**Figure S6**. (a) EDS elemental mapping of (MoFeCoNi)S_x_ displays a uniform distribution of constituents (b) Corresponding EDS spectra validate the precise stoichiometric composition and accurate atomic ratios of its constituent elements, including molybdenum, iron, cobalt, nickel, and sulfur.

**Figure S7**. (a-c) SEM images of (CoNiMo)S_x_ at different magnifications.

**Figure S8**. (a) EDS elemental mapping of (CoNiMo)S_x_ displays a uniform distribution of constituents (b) Corresponding EDS spectra validate the precise stoichiometric composition and accurate atomic ratios of its constituent elements, including molybdenum, cobalt, nickel, and sulfur.


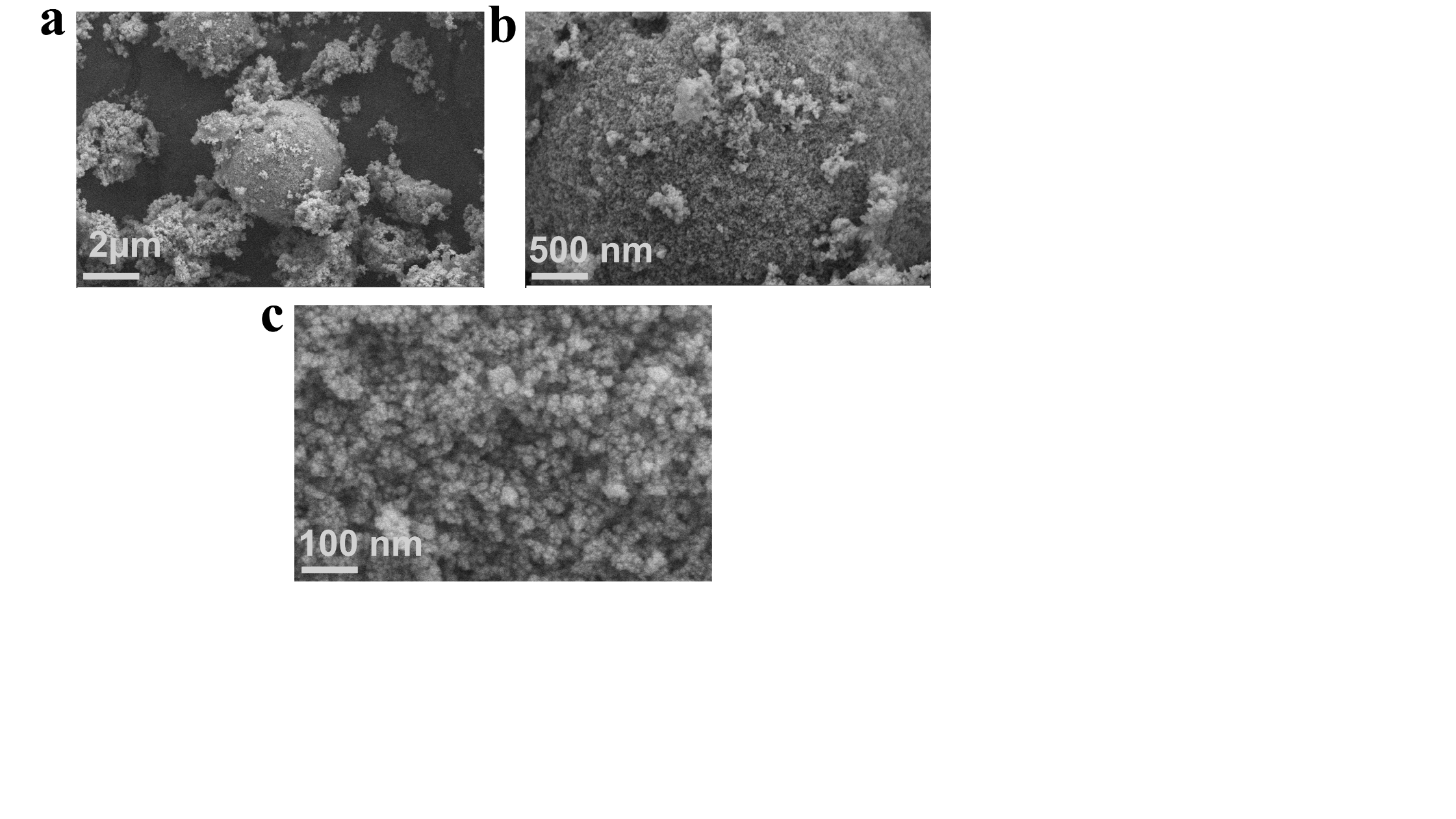


**Figure S9**. (a-c) SEM images of (FeNiMo)S_x_ at different magnifications.

**Figure S10**. (a) EDS elemental mapping of (FeNiMo)S_x_ displays a uniform distribution of constituents (b) Corresponding EDS spectra validate the precise stoichiometric composition and accurate atomic ratios of its constituent elements, including molybdenum, iron, nickel, and sulfur.

**Figure S11**. XPS survey scan of (a) (VMoFeCoNi)S_x_, (b) (MoFeCoNi)S_x_, (c) (CoNiMo)S_x_, and (d) (FeNiMo)S_x_

**Figure S12**. Linear sweep voltammetry (LSV) curves for OER of (VMoFeCoNi)S_x_/NF, IrO_2_/NF, and bare NF, highlighting the superior performance of HEMC over benchmark catalyst IrO_2_ and revealing negligible anodic current by NF.

**Figure S13**. Linear sweep voltammetry (LSV) curves for OER of (VMoFeCoNi)S_x_/NF, NiFe/NF, highlighting the superior performance of HEMC over benchmark catalyst NiFe.


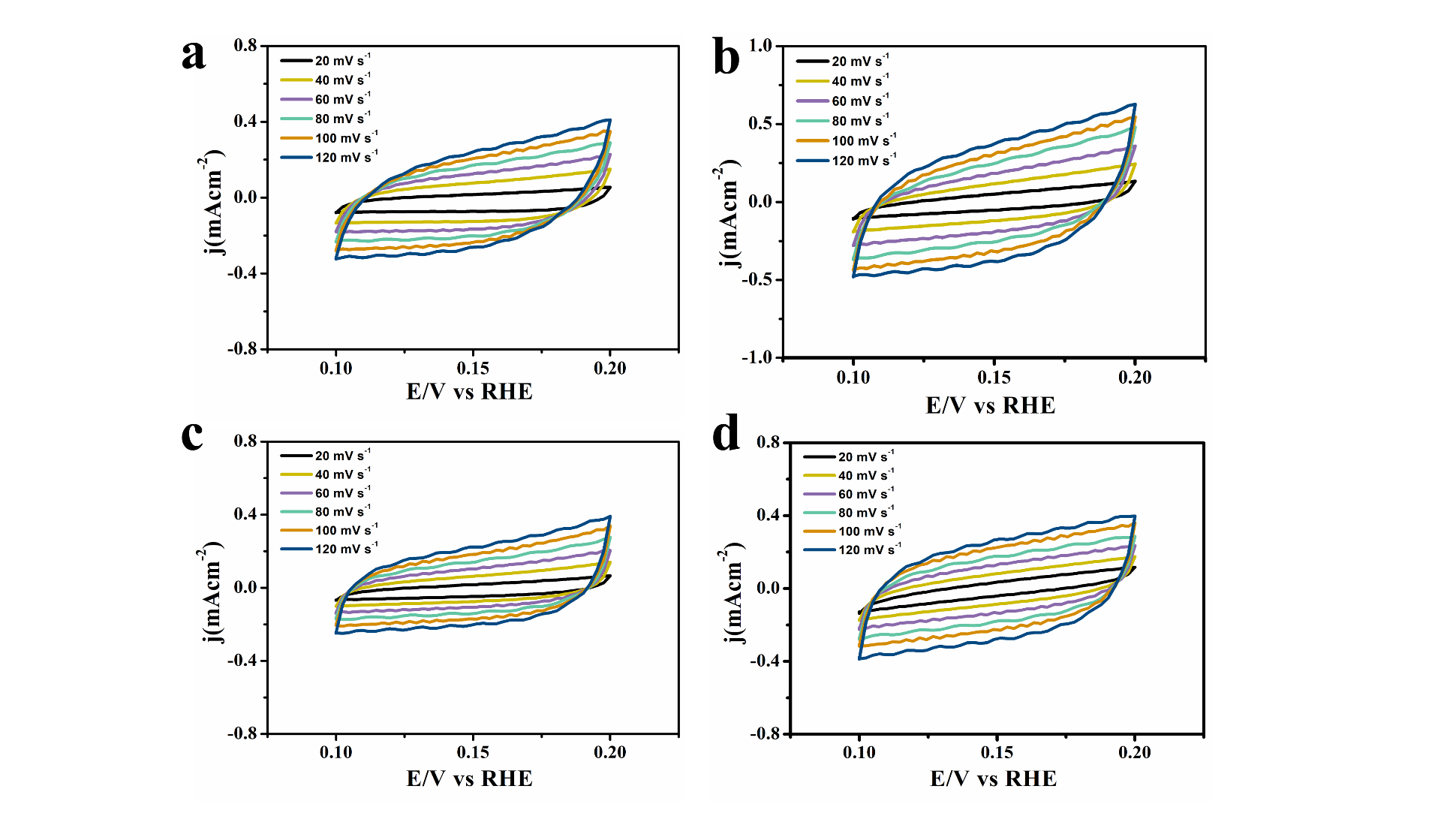


**Figure S14**. Cyclic voltammetry (CV) curves obtained in the non-Faradaic region at varying scan rates for double-layer capacitance (Cdl) measurements of: (a) (VMoFeCoNi)S_x_/NF, (b) (MoFeCoNi)S_x_/NF, (c) (FeNiMo)S_x_/NF, and (d) (CoNiMo)S_x_/NF. The electrochemically active surface area (ECSA) is directly correlated with Cdl, making the Cdl measurement a widely adopted method for quantifying the ECSA of electrocatalysts.

**Figure S15**. Mass activity (Ag^-1^) and exchange current density (mAcm^-2^) evaluated at 1.53 V

**Figure S16**. Illustration of the initial electrochemically active surface area (ECSA)-normalized linear sweep voltammetry (LSV) curves of HEMC and reference samples in a 1.0 M KOH electrolyte. The data reveal that the HEMC exhibits exceptional electrocatalytic performance relative to its counterparts, even after ECSA normalization, underscoring its superior efficiency for the OER.

**Figure S17**. A comparison of the apparent turnover frequency (TOF) of the HEMC and its reference samples at a potential of 1.50 V demonstrates the superior intrinsic electrocatalytic activity of the HEMC.

**Figure S18**. Faradaic efficiency (FE) measurements of HEMC and its reference samples. The data reveal that the HEMC exhibits high FE, which closely matches the theoretical values relative to its counterparts, underscoring that the current during the electrochemical process primarily originates from oxygen evolution.

**Figure S19**. Comparative chronopotentiometry test at 200 mA cm^-2^ of (a) (MoFeCoNi)S_x_, (b) (CoNiMo)S_x_, and (c) (FeNiMo)S_x_.

**Figure S20**. LSV polarization curves after 120 h of prolonged stability at 200 mA cm^-2^, highlighting the activity and stability of HEMC.

**Figure S21**. (a, b) SEM images at different magnifications of the HEMC after 120 h stability at 200 mA cm^-2,^ revealing minor macroscopic surface cracks but preservation of the characteristic nanoflake morphology

**Figure S22**. EDS elemental mapping and Corresponding EDS spectra of the HEMC after 120 h stability at 200 mA cm^-2^, revealing elemental retention.

**Figure S23**. XRD pattern of HEMC before and after 120 h stability at 200 mA cm^-2^, showing no significant changes, confirming that the HEMC maintained its pristine crystallographic structure. The good stability of HEMC is attributed to the high-entropy nature to stabilize its phase structure.


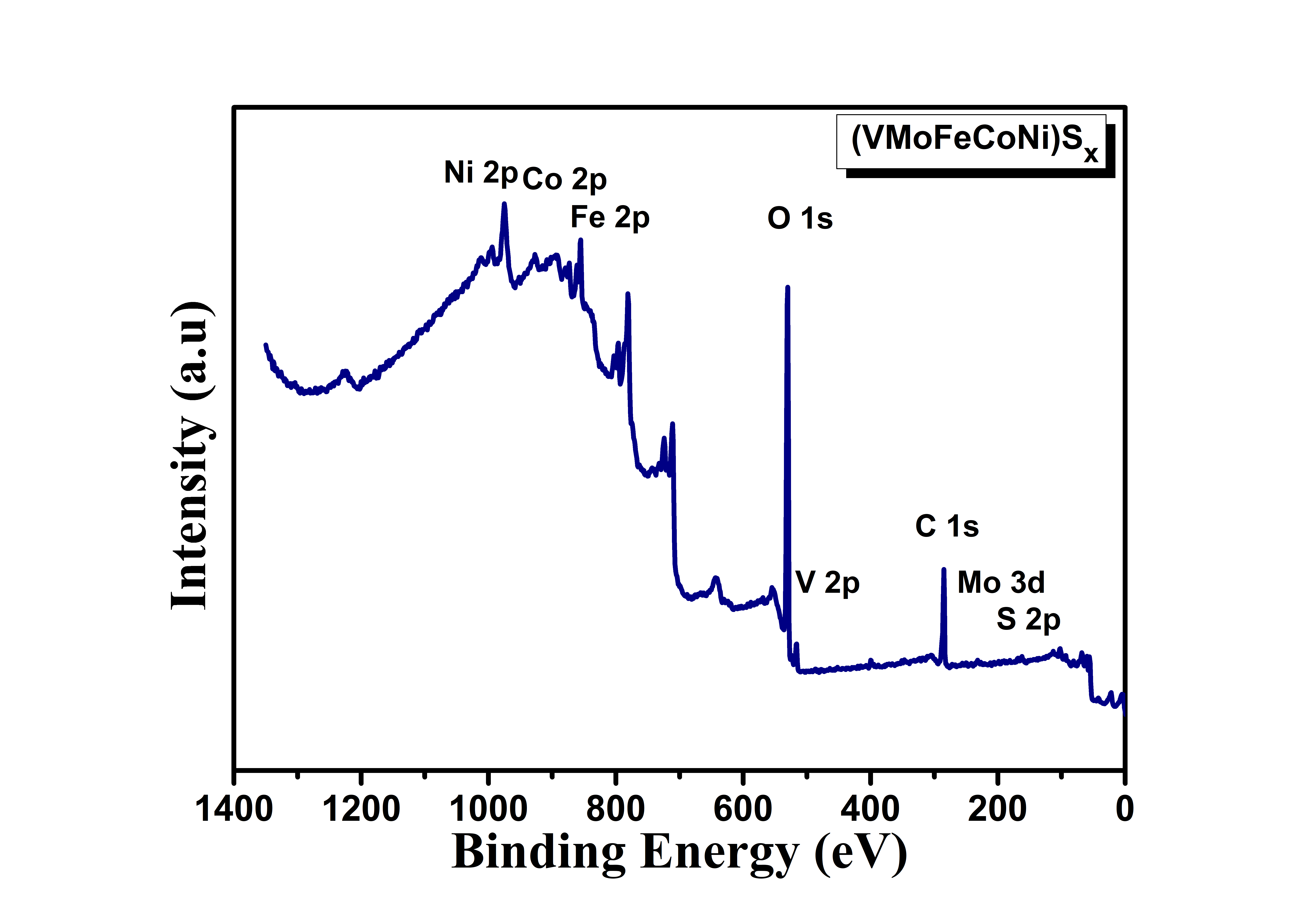


**Figure S24**. XPS survey of HEMC after 120 h of stability test, confirming the presence of V, Mo, Fe, Co, Ni, and S

**Figure S25**. High resolution XPS spectra of a) Co 2p, b) Fe 2p, c) Mo 3d, d) Ni 2p, e) O 1s & V 2p, f) S 2p of HEMC after 120 h stability test at 200 mA cm^-2^

**Table S1**: Summary of the crystallite size, dislocation density, micro strain, and Overpotential at 50 mA cm^-2^

| **Material** | **2Ѳ** | **β (º)** | **δ × 10^-3^ (nm^-2^)** | **ɛ × 10^-3^** | **D**  **(nm)** | **Average crystallite size** | **η@50**  **mA cm^-2^** |
| --- | --- | --- | --- | --- | --- | --- | --- |
| (VMoFeCoNi)S_x_ | 30.0 | 0.691 | 5.16 | 0.67%  $\pm0.0018$ | 21.4 | $\pm$**18 nm** | **220** |
|  | 35.3 | 0.733 | 4.39 |  | 15.0 |  |  |
|  | 44.4 | 0.470 | 3.08 |  | 18.0 |  |  |
|  | 51.7 | 0.625 | 5.61 |  | 13.3 |  |  |
|  | 56.9 | 0.644 | 3.43 |  | 17.0 |  |  |
|  | 62.5 | 0.782 | 3.76 |  | 16.2 |  |  |
|  | 76.1 | 0.726 | 2.21 |  | 21.2 |  |  |
| (MoFeCoNi)S_x_ | 30.1 | 0.576 | 4.98 | 0.59%  $\pm$0.00121 | 22.4 | $\pm$**25 nm** | **250** |
|  | 35.5 | 0.636 | 3.74 |  | 16.3 |  |  |
|  | 44.3 | 0.448 | 2.98 |  | 22.4 |  |  |
|  | 51.9 | 1.91 | 3.16 |  | 29.2 |  |  |
|  | 57.0 | 0.530 | 1.13 |  | 29.6 |  |  |
|  | 62.6 | 0.615 | 2.44 |  | 20.2 |  |  |
|  | 73.7 | 0.598 | 0.88 |  | 33.5 |  |  |
| (FeNiMo)S_x_ | 30.9 | 0.678 | 3.27 | 0.46%  $\pm$0.00127 | 17.4 | $\pm$**19 nm** | **260** |
|  | 35.7 | 0.613 | 2.01 |  | 12.8 |  |  |
|  | 44.3 | 0.410 | 2.66 |  | 19.3 |  |  |
|  | 49.9 | 0.787 | 3.97 |  | 12.9 |  |  |
|  | 57.1 | 1.76 | 3.91 |  | 15.9 |  |  |
|  | 62.6 | 0.786 | 3.44 |  | 17.0 |  |  |
|  | 76.1 | 0.718 | 1.02 |  | 31.1 |  |  |
| (CoNiMo)S_x_ | 31.1 | 0.678 | 3.14 | 0.38%  $\pm$0.00138 | 31.5 | $\pm$**31 nm** | **310** |
|  | 35.4 | 0.593 | 2.37 |  | 20.5 |  |  |
|  | 44.3 | 0.401 | 4.01 |  | 14.1 |  |  |
|  | 51.5 | 2.797 | 3.48 |  | 16.9 |  |  |
|  | 56.3 | 2.930 | 2.88 |  | 18.6 |  |  |
|  | 61.4 | 0.716 | 1.86 |  | 23.1 |  |  |
|  | 76.1 | 0.798 | 3.58 |  | 41.2 |  |  |

2Ѳ = Peak position, β = FWHM, δ = Dislocation density, ɛ = Micro strain, D = Crystallites size

| **Material** | **Mode** | **Peak position** | **Δν (vs. [Standard], cm^-1^)** | **FWHM (cm^-1^)** | **R^2^** | **References** |
| --- | --- | --- | --- | --- | --- | --- |
| (VMoFeCoNi)S_x_ | E_g_ | 467.8 | $\pm$132.8 vs. FeS_2_  $\pm$175.8 vs. CoS_2_ | 52.8 | 0.9892 | [^1^]  [^2^] |
|  | A_1g_ | 554.1 | $\pm$187.1 vs. FeS_2_  $\pm$164.1 vs. CoS_2_ | 83.8 | 0.9981 |  |
| (MoFeCoNi)S_x_ | E_g_ | 459.3 | $\pm$124.3 vs. FeS_2_  $\pm$167.3 vs. CoS_2_ | 52.3 | 0.9897 |  |
|  | A_1g_ | 545.1 | $\pm$178.1 vs. FeS_2_  $\pm$155.1 vs. CoS_2_ | 79.3 | 0.9983 |  |
| (FeNiMo)S_x_ | E_g_ | 468.9 | $\pm$133.9 vs. FeS_2_  $\pm$176.9 vs. CoS_2_ | 51.9 | 0.9326 |  |
|  | A_1g_ | 543.5 | $\pm$176.5 vs. FeS_2_  $\pm$153.5 vs. CoS_2_ | 73.0 | 0.9813 |  |
| (CoNiMo)S_x_ | E_g_ | 455.1 | $\pm$120.1 vs. FeS_2_  $\pm$163.1 vs. CoS_2_ | 49.2 | 0.9164 |  |
|  | A_1g_ | 510.7 | $\pm$143.7 vs. FeS_2_  $\pm$120.7 vs. CoS_2_ | 67.6 | 0.9879 |  |

**Table S2**: Summary of the characteristic’s vibrational modes, Δν, FWHM and fitting quality (R^2^)

**Table S3**: Comparison of OER performance among the reported High-entropy sulfide catalysts in alkaline media.

| **Electrocatalyst** | **Synthetic description** | **η@10 mA cm^-2^** | **Tafel slope (mVdec^-1^)** | **ECSA (mF cm^-2^)** | **References** |
| --- | --- | --- | --- | --- | --- |
| (FeCoNiMn)S_2_ | Annealing | 200 | 40.5 | 18.5 | [^3^] |
| (FeCoNiCrCuAl)S@HCS | Solvothermal | 253 | 61.5 | 0.67 | [^4^] |
| H-FeCoS@NC | Annealing | 240 | 81.3 | 17.6 | [^5^] |
| FeNiCoCrMnS_2_ | Solvothermal | 199 | 39.1 | 0.59 | [^6^] |
| (MnFeCoNiCu)S_2_ | Solvothermal | 221 | 54.4 | 6.85 | [^7^] |
| (CrMnFeCoNi)S_x_ | Pulse thermal decomposition | 295@η_100_ | 66 | 84 | [^8^] |
| (NiFeCoMn)_3_S_4_ | Solvothermal | 289 | 75.6 | 4.5 | [^9^] |
| CoZnCdCuMnS@CF | Hydrothermal | 173 | 69.8 | 6.4 | [^10^] |
| **(VMoFeCoNi)S_x_** | **Solvothermal** | **η_50_ 210** | **66** | **29.7** | **This work** |

**Table S4**: Comparison of atomic percentages of elements in HEMC before and after OER stability test

| **Element** | **Atomic percentage (At%)** | |
| --- | --- | --- |
|  | **Before** | **After** |
| V | 11.6 | 10.3 |
| Ni | 5.9 | 6.7 |
| Co | 5.6 | 6.8 |
| Fe | 6.4 | 7.6 |
| Mo | 6.2 | 6.0 |
| S | 64.3 | 62.1 |

**Table S5**: Summary of quantitative pre-OER and post-OER XPS data of HEMC

| **Element** | **Component peak** | **Pre-OER (BE,** $\boldsymbol{\pm}$**0.1** **eV)** | **Pre-OER (relative%)** | **Post-OER (BE,** $\boldsymbol{\pm}$**0.1eV)** | **Post-OER (relative%)** | **ΔBE (eV)** |
| --- | --- | --- | --- | --- | --- | --- |
| Co 2p | Co^+3^ 2p_3/2_ | 780.5 | 46.80 | 781.0 | 55.30 | +0.5 |
|  | Co^+2^ 2p_3/2_ | 782.1 |  | 783.6 |  | +1.5 |
| Fe 2p | Fe^+2^ 2p_3/2_ | 711.4 | 52.83 | 712.0 | 63.02 | +0.6 |
|  | Fe^+3^ 2p_3/2_ | 714.4 |  | 714.9 |  | +0.5 |
| Mo 3d | Mo^+6^ 3d_5/2_ | 232.0 | 70.49 | 232.4 | 72.50 | +0.4 |
| Ni 2p | Ni^+2^ 2p_3/2_ | 855.3 | 60.08 | 855.6 | 64.87 | +0.3 |
|  | Ni^+3^ 2p_3/2_ | 856.5 |  | 856.7 |  | +0.2 |
| V 2p | V^+5^ 2p_3/2_ | 516.7 | 18.34 | 516.7 | 7.49 | 0 |

**References**

1. Sha, R.; Kadu, A.; Matsumoto, K.; Uno, S.; Badhulika, S., Ultra-low cost, smart sensor based on pyrite FeS2 on cellulose paper for the determination of vital plant hormone methyl jasmonate. *Engineering Research Express* **2020,** *2* (2), 025020.

2. Ma, D.; Hu, B.; Wu, W.; Liu, X.; Zai, J.; Shu, C.; Tadesse Tsega, T.; Chen, L.; Qian, X.; Liu, T. L., Highly active nanostructured CoS2/CoS heterojunction electrocatalysts for aqueous polysulfide/iodide redox flow batteries. *Nature Communications* **2019,** *10* (1), 3367.

3. Cai, H.; He, S.; Yang, H.; Huang, Q.; Luo, F.; Hu, Q.; Zhang, X.; He, C., Highly Exposed Ultra‐Small High‐Entropy Sulfides with d‐p Orbital Hybridization for Efficient Oxygen Evolution. *Advanced Materials* **2025,** *37* (33), 2508610.

4. Wan, Y.; Wei, W.; Ding, S.; Wu, L.; Yuan, X., Achieving Efficient Oxygen Evolution on High‐Entropy Sulfide Utilizing Low Electronegativity of Al. *Small* **2024,** *20* (46), 2404689.

5. Li, X.; Zhang, M.; Liu, Y.; Sun, X.; Li, D.; Liu, B.; Yang, M.; Chen, H.; Ding, S.; Lin, Z., Highly efficient and durable water electrolysis via ligand modulated interfacial assembly. *Applied Catalysis B: Environment and Energy* **2024,** *359*, 124530.

6. Nguyen, T. X.; Su, Y. H.; Lin, C. C.; Ting, J. M., Self‐reconstruction of sulfate‐containing high entropy sulfide for exceptionally high‐performance oxygen evolution reaction electrocatalyst. *Advanced Functional Materials* **2021,** *31* (48), 2106229.

7. Li, F.; Ma, Y.; Wu, H.; Zhai, Q.; Zhao, J.; Ji, H.; Tang, S.; Meng, X., Sub-3-nm high-entropy metal sulfide nanoparticles with synergistic effects as promising electrocatalysts for enhanced oxygen evolution reaction. *The Journal of Physical Chemistry C* **2022,** *126* (43), 18323-18332.

8. Cui, M.; Yang, C.; Li, B.; Dong, Q.; Wu, M.; Hwang, S.; Xie, H.; Wang, X.; Wang, G.; Hu, L., High‐entropy metal sulfide nanoparticles promise high‐performance oxygen evolution reaction. *Advanced Energy Materials* **2021,** *11* (3), 2002887.

9. Wu, L.; Shen, X.; Ji, Z.; Yuan, J.; Yang, S.; Zhu, G.; Chen, L.; Kong, L.; Zhou, H., Facile synthesis of medium‐entropy metal sulfides as high‐efficiency electrocatalysts toward oxygen evolution reaction. *Advanced Functional Materials* **2023,** *33* (3), 2208170.

10. Lei, Y.; Zhang, L.; Xu, W.; Xiong, C.; Chen, W.; Xiang, X.; Zhang, B.; Shang, H., Carbon-supported high-entropy Co-Zn-Cd-Cu-Mn sulfide nanoarrays promise high-performance overall water splitting. *Nano Research* **2022,** *15* (7), 6054-6061.
